# Reconstructing high-order SERS transitions in an overlooked spectral region reveals surface complex formation

**DOI:** 10.1038/s42004-026-02136-x

**Published:** 2026-07-17

**Authors:** Ivan Kopal, Matěj Kmetík, Pavel Matějka, Marek Piliarik, Marcela Dendisová

**Affiliations:** 1 Department of Physical Chemistry, University of Chemistry and Technology Prague, Technická 5, Prague 6, Czech Republic; 2 Institute of Photonics and Electronics, Czech Academy of Sciences, Chaberská 1014/57, Prague 8, Czech Republic; 3 Institute of Organic Chemistry and Biochemistry, Czech Academy of Sciences, Flemingovo náměstí 542/2, Prague 6, Czech Republic

**Keywords:** Raman spectroscopy, Photocatalysis, SERS

## Abstract

The complexity of information encoded in surface-enhanced Raman scattering (SERS) spectra provides both tremendous opportunities and challenges for molecular characterization. While conventional SERS studies focus on the spectral window below 2000 cm⁻¹, high-order transitions, including overtones and combination bands above this region, have received limited attention despite their potential to reveal detailed insights into molecular surface interactions. Here, we investigate these high-order transitions using 4-aminobenzenethiol (4-ABT) as a model system, which undergoes photon-induced chemical transformations and lacks electronic transitions in the visible range. SERS spectra were recorded at excitation wavelengths of 455, 532, 633, and 780 nm on highly enhancing Ag substrate, and theoretical spectra were constructed from fundamental bands to assign the high-order features. The results demonstrate that the observed high-order bands in these systems originate exclusively from 4,4′-dimercaptoazobenzene (4,4′-DMAB) formed on the surface, with excitation-wavelength dependence reflecting the number of dimers and surface complex formation. Control experiments on Au and Cu substrates and with other molecules confirm the generality of these observations and rule out instrumental artifacts. These findings establish high-order SERS transitions as reliable markers of molecule–metal surface complexes and provide a broader spectral perspective for analytical and fundamental SERS studies.

**Figure:**
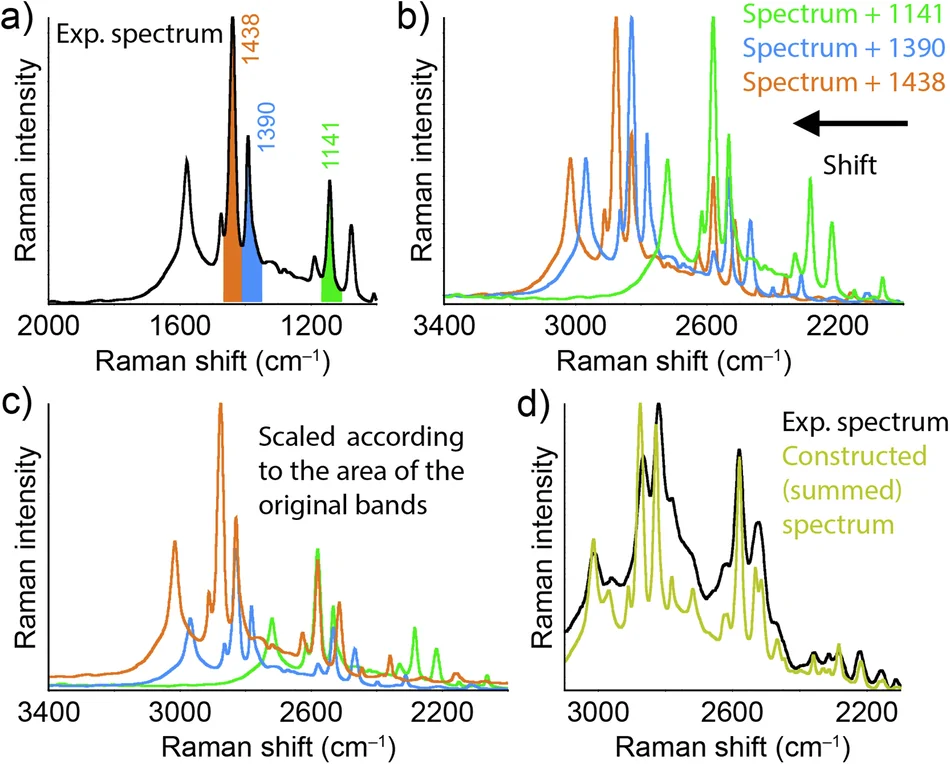

## Abstract (web summary)

Conventional surface-enhanced Raman scattering (SERS) studies focus on the spectral window below 2000 cm⁻¹, while high-order transitions have received limited attention despite their potential to reveal detailed insights into molecular surface interactions. Here, the authors study such high-order transitions using 4-aminobenzenethiol (4-ABT) as a model system, showing that they are a direct spectroscopic marker of molecule-metal surface complex formation and originate from 4,4′-dimercaptoazobenzene (4,4′-DMAB) photochemically formed on the surface.

## Introduction

The complexity of information encrypted in the overall spectral response of surface-enhanced Raman scattering (SERS) provides the most tremendous benefits and severe challenges when aiming for any of the wide range of applications^1,2^. This effect, providing enormous enhancement of the probed molecules’ signal, results from several enhancing mechanisms in close distances from the plasmonic surfaces^3,4^. Among them, the electromagnetic mechanism is widely considered to be the one majorly contributing to the overall amplification of the signal through the resonance of both incident and scattered light with plasmon resonant frequencies, and therefore strongly affected through the optimization of nanofeatures’ morphology and material^5,6^. However, the appropriate molecular orbital distribution with respect to the energetic levels of plasmonic metal may increase the signal by several orders of magnitude, and at the same time, change the appearance of recorded spectra^7–9^. Furthermore, some molecules are capable of forming covalent bonds with surface metal atoms, thereby altering their geometry and electronic properties^10,11^. These so-called “surface complexes” are subsequently not rarely prone to undergo another transformation because of interaction with incidental photons^12^. Therefore, the exact determination of the species from which the signal arises is not straightforward^13^. Furthermore, when dealing with more complex samples, the assignment procedure may become even more challenging as several probed molecules (potentially several species of each molecule) would be present on the surface^12,14^.

In some cases, this issue may prove insurmountable, particularly in samples that contain several molecules with similar structural motives, resulting in closely overlapping bands^12^. This problem is further highlighted by the fact that SERS research generally concentrates solely on a restricted spectral window, rarely extending beyond 2000 cm⁻^1^. Although this interval encompasses the vast majority of the vibrational transitions occurring, researchers would often welcome the opportunity to explore additional spectral regions to support their scientific hypotheses more thoroughly. For example, attention may be paid to low-frequency vibrations, where SERS spectroscopy enables access to molecular and electronic excitations by confining visible light to deep sub-wavelength scales^15^. Meanwhile, in conventional vibrational spectroscopy, the area around the 3000 cm⁻^1^ is frequently measured and discussed, as the stretching vibrations containing hydrogen can be found in this region. However, these bands are rarely observed in typical SERS experiments due to their distance from the metal surface, unfavorable orientation, and weak enhancement^3,16,17^. Usually, overtones or combination bands may occur in this region, which are very weak in conventional Raman spectroscopy. For overtones and combination bands in this region, no intrinsic weakening is expected, since their orientation and distance from the metallic surface are the same as for the corresponding fundamentals. The only potential limitation is detuning from the excitation wavelength, which can reduce resonance enhancement, but this effect already applies to the fundamental transitions themselves. Nevertheless, a sufficiently strong overall response is required, as these high-order modes are generally less intense than the fundamentals. Despite this, the topic has received limited attention so far, with most studies merely reporting the occasional observation of such features without further analysis^18–21^. Nevertheless, some studies indicate that these high-order transitions are closely associated with the action of chemical enhancement mechanisms^22,23^.

In this work, we present a comprehensive experimental framework for reconstructing and rigorously interpreting high-order transitions in SERS, thereby directly linking these phenomena to fundamental interfacial processes, such as surface complex formation and photon-induced chemical transformations, with significant implications for analytical chemistry. Our approach enables an experimentally driven assessment of the chemical origin of high-order transitions. To this end, 4-aminobenzenethiol (4-ABT) was selected as a model molecule. Unlike in most previously reported studies, this compound does not exhibit an electronic transition in the visible spectral region, while at the same time being well known for its pronounced photon-induced reactivity. This unique combination enables the investigation of high-order transitions in photon-induced chemical transformations at metal–molecule interfaces. To capture the full nature of these effects, SERS spectra were recorded using excitation wavelengths spanning a wide spectral interval (455, 532, 633, and 780 nm). To ensure sufficient signal intensity for the detailed analysis of subtle spectral changes, we employed large-scale electrochemically prepared substrates with intrinsically high enhancement efficiencies. Our results demonstrate that high-order SERS transitions provide a direct spectroscopic marker of molecule-metal surface complex formation and reveal that their emergence is governed predominantly by chemical enhancement mechanisms associated with electronic transitions within the resulting surface complexes. Importantly, the presented approach enables direct experimental characterization of surface-complex formation and associated chemical transformations, providing molecular-level insight that is often obtained primarily through advanced density functional theory (DFT) calculations.

## Results and discussion

### Reconstruction and assignment of high-order SERS transitions

The behavior of 4-ABT in relation to plasmonic structures and SERS has been widely studied and discussed for several decades. This frequently employed model probe has attracted considerable attention due to the occasional appearance of several bands that are absent in normal Raman spectra (Fig. S1) and therefore cannot be directly assigned to vibrational transitions of the original molecule. Among these so-called “alien bands,” the vibrations at ~1141, 1390, and 1430 cm⁻¹ are the most intense, although additional evidence of others can be found, for example, as distortions of nearby bands^12^. Several theories have been proposed to explain their origin, with two being particularly prominent: (i) extraordinary enhancement through the charge-transfer mechanism, enabled by the nearly ideal alignment of molecular orbitals with the Fermi level of the most commonly used plasmonic metals (Au, Ag, and Cu); and (ii) photon-induced transformation into the dimer 4,4´-dimercaptoazobenzene (4,4´-DMAB), whose calculated spectra show remarkable agreement with the experimentally observed alien bands. Thus, while a significant contribution from chemical enhancement is likely, the presence of these bands strongly suggests transformation of the original molecules into different species. Furthermore, the photoreaction may not proceed in a straightforward manner, leading to the coexistence of multiple 4-ABT-derived species (4-ABTs) on the surface, as also supported by tip-enhanced Raman spectroscopy (TERS) studies, which revealed the presence of both 4-ABT and its dimerization product, along with additional vibrational features attributed to field-gradient effects^24–26^. As a result, many recorded SERS bands may represent convolutions of similar vibrations originating from different species, giving rise to broadened features and complicating the interpretation of 4-ABT's spectra. This ambiguity, therefore, constitutes a key challenge for the reliable use of 4-ABT as a model analyte in SERS studies.

**Fig. 1:**
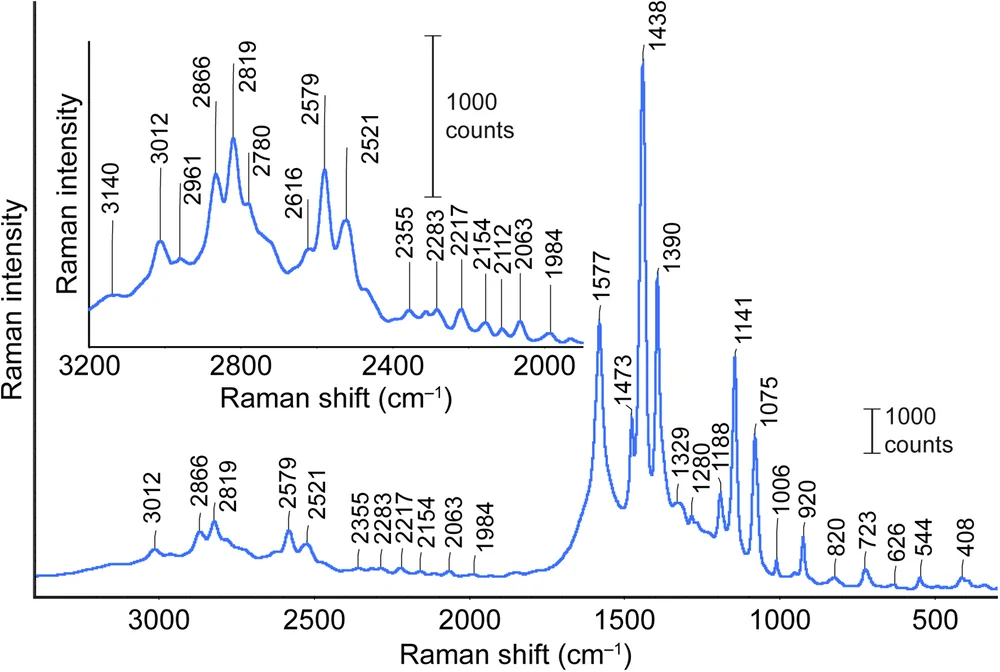
Ag-SERS spectrum of 4-ABT/4,4′-DMAB. Spectrum measured with 455 nm excitation and obtained by averaging 10 individual spectra.

The 4-ABT spectrum measured with the excitation wavelength of 455 nm is shown in Fig. 1. The additional bands discussed in the previous paragraph (namely at 1141, 1390, and 1438 cm⁻¹) are clearly observable, providing evidence of an ongoing reaction on the silver surface. The most important bands of the original monomeric 4-ABT, at 1188, 1075, and 1577 cm⁻¹, are also present, although the latter two have positions similar to bands of other 4-ABT species, which makes them difficult to use for unambiguous identification of surface species. Furthermore, the high-Raman-shift region, highlighted in the Fig. 1 inset, contains numerous bands, with the most intense observed between 3000 and 2500 cm⁻¹. The overall similarity in band patterns with the commonly studied 1600–1000 cm⁻¹ region is apparent, particularly when comparing the relative intensities of bands at 3012, 2866, 2819, 2579, and 2521 cm⁻¹ with those at 1577, 1438, 1390, 1141, and 1075 cm⁻¹. The latter group of bands is typically attributed to the formation of 4,4´-DMAB. At the same time, it is difficult to assign the higher-shift bands to fundamental transitions, as most of them appear in positions inconsistent with expected vibrations of either 4-ABT or 4,4´-DMAB structural motifs. Therefore, high-order transitions represent the most probable explanation. However, these higher-shift vibrations cannot be reproduced simply by multiplying the lower Raman shifts by a common factor, indicating that the observed features are not solely overtones. In contrast, they can be obtained by adding ~1440 cm⁻¹ to the Raman shifts of the lower region, supporting their interpretation as combination transitions involving the related fundamentals. It should be noted that although aromatic molecules typically exhibit distinctive –CH stretching modes in the ~3000 cm⁻¹ region in conventional Raman spectroscopy, these vibrations are often weak in SERS due to their distance from the metal surface, unfavorable orientation, and lower enhancement efficiency. Therefore, any contribution of –CH stretching fundamentals to the spectra presented here is expected to be minor and may only manifest as a slight background in the high-order transition region.

**Fig. 2:**
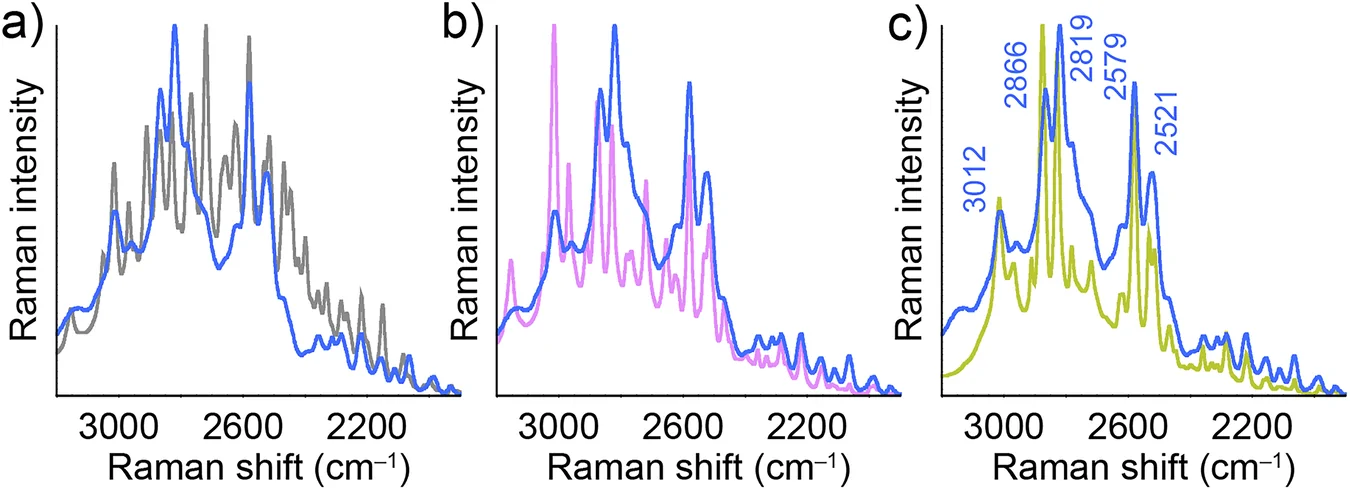
Reconstruction of high-order SERS transitions. Comparison of spectra constructed from: **a** the sum of all spectra shifted to higher Raman shift values according to individual band positions; **b** the sum of all spectra shifted to higher Raman shift values according to individual band positions and scaled according to the area of the original bands; **c** the sum of spectra shifted to higher Raman shift values of 1141, 1390, and 1438 cm⁻¹ and scaled according to the area of the original bands. Each constructed spectrum is compared with the experimental spectrum (blue) measured with 455 nm excitation in the 3200–1900 cm⁻¹ region. The spectra are displayed on a full-scale basis.

We developed an approach to reconstruct high-order spectra by combining the most intense fundamental Raman bands with each other. In practice, this involved taking the measured fundamental spectrum and, for each fundamental band, shifting the x-axis (Raman shift) by the value of that band, thereby creating a set of “combination spectra” in which each band is combined with every other, including itself (simulating overtones). From these, we then attempted to construct an overall spectrum most similar to that observed experimentally in the 3000–2500 cm⁻¹ region (Fig. 2 and Fig. S2). As a first step, we averaged all spectra with equal weight, yielding the spectrum shown in Fig. 2a. While the positions of the constructed bands agreed with the experiment, the relative band areas differed significantly. Therefore, in the second step, we calculated a weighted average, where the weight corresponded to the area of the fundamental band used for shifting. This approach is reasonable, as the probability of observing high-order transitions increases with the intensity of the fundamental. The resulting spectrum (Fig. 2b) was more consistent with the experiment, although discrepancies in relative areas, particularly at higher Raman shifts, remained. In the final step, we performed a weighted average using only selected “combination spectra.” Although all fundamental bands were initially considered, the best agreement was achieved when using spectra shifted by 1438, 1390, and 1141 cm⁻¹, corresponding to the most intense modes of the 4,4´-DMAB species. (Fig. 2c). This procedure shows that the constructed spectra capture the main experimental high-order features and provide a systematic basis for their interpretation. The resulting spectra were therefore used for the vibrational assignment of high-order transitions (Table 1). Notably, this procedure involved only bands typically attributed to the 4,4´-DMAB molecule; thus, the assignment of fundamental transitions of 4,4´-DMAB served as the basis (Table S1)^12,13^. As expected, high-order transitions consist of both overtones and combination transitions, with the latter generally being more intense. In the 455-nm excited spectrum, the overtones of 1438, 1390, and 1141 cm⁻¹ are found at 2866, 2780, and 2283 cm⁻¹, respectively. Among the combination bands, the most pronounced are observed at 3012 cm⁻¹ (1577 + 1438 cm⁻¹), 2818 cm⁻¹ (1438 + 1390 cm⁻¹), 2579 cm⁻¹ (1438 + 1141 cm⁻¹), and 2521 cm⁻¹ (1390 + 1141 cm⁻¹ and/or 1438 + 1075 cm⁻¹). This agrees with the relative areas of the fundamentals, as these same bands are among the most intense and therefore give rise to the strongest combinations and overtones. The difference between the Raman shifts of the constructed bands and the experimental values does not exceed 10 cm⁻¹, which can be fully explained by anharmonicity-related energy differences not considered in our construction procedure. Importantly, the fact that the experimental shape can be reconstructed exclusively from 4,4´-DMAB bands implies that this molecule is solely responsible for the observed high-order vibrations. Consequently, this spectral region can be reliably used in cases where potential contamination by other ABT species must be excluded, both for addressing fundamental questions and for precise analytical evaluations.

**Table 1:** Vibrational assignment of high-order transition modes of 4,4´-DMAB

Raman shift (cm^−1^)			Assignment of the vibrational modes	
Experimental^a^	Constructed^b^	*ΔX*	Combination transition (C) / Overtone (O)	[Vibration]_Y_
3012	3014	−2	C	[ν (C–C)_Ar_, ν (C = C)_Ar_, δ_ip_ (C–H)_Ar_]_1577_ + [ν (N = N), ν (C–C)_Ar_, ν (C = C)_Ar_, δ_ip_ (C–H)_Ar_]_1438_
2961	2969	−8	C	[ν (C–C)_Ar_, ν (C = C)_Ar_, δ_ip_ (C–H)_Ar_]_1577_ + [ν (N = N), ν (C–C)_Ar_, ν (C = C)_Ar_, δ_ip_ (C–H)_Ar_]_1390_
	2910		C	[δ_ip_ (C–H)_Ar_]_1473_ + [ν (N = N), ν (C–C)_Ar_, ν (C = C)_Ar_, δ_ip_ (C–H)_Ar_]_1438_
2866	2875	−9	O	[ν (N = N), ν (C–C)_Ar_, ν (C = C)_Ar_, δ_ip_ (C–H)_Ar_]_1438_
2819	2828	−9	C	[ν (N = N), ν (C–C)_Ar_, ν (C = C)_Ar_, δ_ip_ (C–H)_Ar_]_1438_ + [ν (N = N), ν (C–C)_Ar_, ν (C = C)_Ar_, δ_ip_ (C–H)_Ar_]_1390_
2780	2781	−1	O	[ν (N = N), ν (C–C)_Ar_, ν (C = C)_Ar_, δ_ip_ (C–H)_Ar_]_1390_
2720	2718	+2	C	[ν (C–C)_Ar_, ν (C = C)_Ar_, δ_ip_ (C–H)_Ar_]_1577_ + [ν (C–N), δ_ip_ (C–H)_Ar_]_1141_ / [ν (N = N), ν (C–C)_Ar_, ν (C = C)_Ar_, δ_ip_ (C–H)_Ar_]_1438_ + [δ_ip_ (C–H)_Ar_]_1280_
2626	2624	+2	C	[ν (N = N), ν (C–C)_Ar_, ν (C = C)_Ar_, δ_ip_ (C–H)_Ar_]_1438_ + [ν (C–N), δ_ip_ (C–H)_Ar_]_1188_
2616	2616	0	C	[δ_ip_ (C–H)_Ar_]_1473_ + [ν (C–N), δ_ip_ (C–H)_Ar_]_1141_
2579	2579	0	C	[ν (N = N), ν (C–C)_Ar_, ν (C = C)_Ar_, δ_ip_ (C–H)_Ar_]_1438_ + [ν (C–N), δ_ip_ (C–H)_Ar_]_1141_
2521	2531	−10	C	[ν (N = N), ν (C–C)_Ar_, ν (C = C)_Ar_, δ_ip_ (C–H)_Ar_]_1390_ + [ν (C–N), δ_ip_ (C–H)_Ar_]_1141_
	2514	+7	C	[ν (N = N), ν (C–C)_Ar_, ν (C = C)_Ar_, δ_ip_ (C–H)_Ar_]_1438_ + [ν (C–S)]_1075_
2469	2466	+3	C	[ν (N = N), ν (C–C)_Ar_, ν (C = C)_Ar_, δ_ip_ (C–H)_Ar_]_1390_ + [ν (C–S)]_1075_
	2445		C	[ν (N = N), ν (C–C)_Ar_, ν (C = C)_Ar_, δ_ip_ (C–H)_Ar_]_1438_ + [δ_ip_ (C–H)_Ar_]_1006_
2396	2397	−1	C	[ν (N = N), ν (C–C)_Ar_, ν (C = C)_Ar_, δ_ip_ (C–H)_Ar_]_1390_ + [δ_ip_ (C–H)_Ar_]_1006_
2355	2358	−3	C	[ν (N = N), ν (C–C)_Ar_, ν (C = C)_Ar_, δ_ip_ (C–H)_Ar_]_1438_ + [δ_ip_ (C–N = N–C)]_920_
	2329		C	[ν (C–N), δ_ip_ (C–H)_Ar_]_1188_ + [ν (C–N), δ_ip_ (C–H)_Ar_]_1141_ / [ν (N = N), ν (C–C)_Ar_, ν (C = C)_Ar_, δ_ip_ (C–H)_Ar_]_1390_ + [δ_oop_ (C–H)_Ar_]_947_
2312	2311	+1	C	[ν (N = N), ν (C–C)_Ar_, ν (C = C)_Ar_, δ_ip_ (C–H)_Ar_]_1390_ + [δ_ip_ (C–N = N–C)]_920_
2283	2282	+1	O	[ν (C–N), δ_ip_ (C–H)_Ar_]_1141_
2217	2216	+1	C	[ν (C–N), δ_ip_ (C–H)_Ar_]_1141_ + [ν (C–S)]_1075_
2154	2162	−8	C	[ν (N = N), ν (C–C)_Ar_, ν (C = C)_Ar_, δ_ip_ (C–H)_Ar_]_1438_ + [δ_oop_ (C–N), ν (C–C)_Ar_, ν (C = C)_Ar_, δ_oop_ (C–H)_Ar_]_723_
	2150	+4	C	[ν (N = N), ν (C–C)_Ar_, ν (C = C)_Ar_, δ_ip_ (C–H)_Ar_]_1438_ + [δ_oop_ (C–N), ν (C–C)_Ar_, ν (C = C)_Ar_, δ_oop_ (C–H)_Ar_]_715_ / [ν (C–N), δ_ip_ (C–H)_Ar_]_1141_ + [δ_ip_ (C–H)_Ar_]_1006_
2112	2113	−1	C	[ν (N = N), ν (C–C)_Ar_, ν (C = C)_Ar_, δ_ip_ (C–H)_Ar_]_1390_ + [δ_oop_ (C–N), ν (C–C)_Ar_, ν (C = C)_Ar_, δ_oop_ (C–H)_Ar_]_723_
2063	2062	+1	C	[ν (C–N), δ_ip_ (C–H)_Ar_]_1141_ + [δ_ip_ (C–N = N–C)]_920_
1984	1981	+3	C	[ν (N = N), ν (C–C)_Ar_, ν (C = C)_Ar_, δ_ip_ (C–H)_Ar_]_1438_ + [δ_oop_ (C–N), ν (C–C)_Ar_, ν (C = C)_Ar_, δ_oop_ (C–H)_Ar_]_544_
1933	1939	−6	C	[ν (N = N), ν (C–C)_Ar_, ν (C = C)_Ar_, δ_ip_ (C–H)_Ar_]_1390_ + [δ_oop_ (C–N), ν (C–C)_Ar_, ν (C = C)_Ar_, δ_oop_ (C–H)_Ar_]_544_

*a*–455-nm excitation, *b*–constructed from the 455-nm excited spectra using the procedure described in Fig. S1, *ΔX*–difference between experimental and constructed value, *C*–combination transition; *O*–overtone, *Y*–Raman shift of the corresponding fundamental transition, *ν*–stretching, *δ*–deformation, *ip*–in-plane, oop–out-of-plane; *Ar*–aromatic ring.

### Excitation wavelength dependence and origin of high-order SERS transitions

**Fig. 3:**
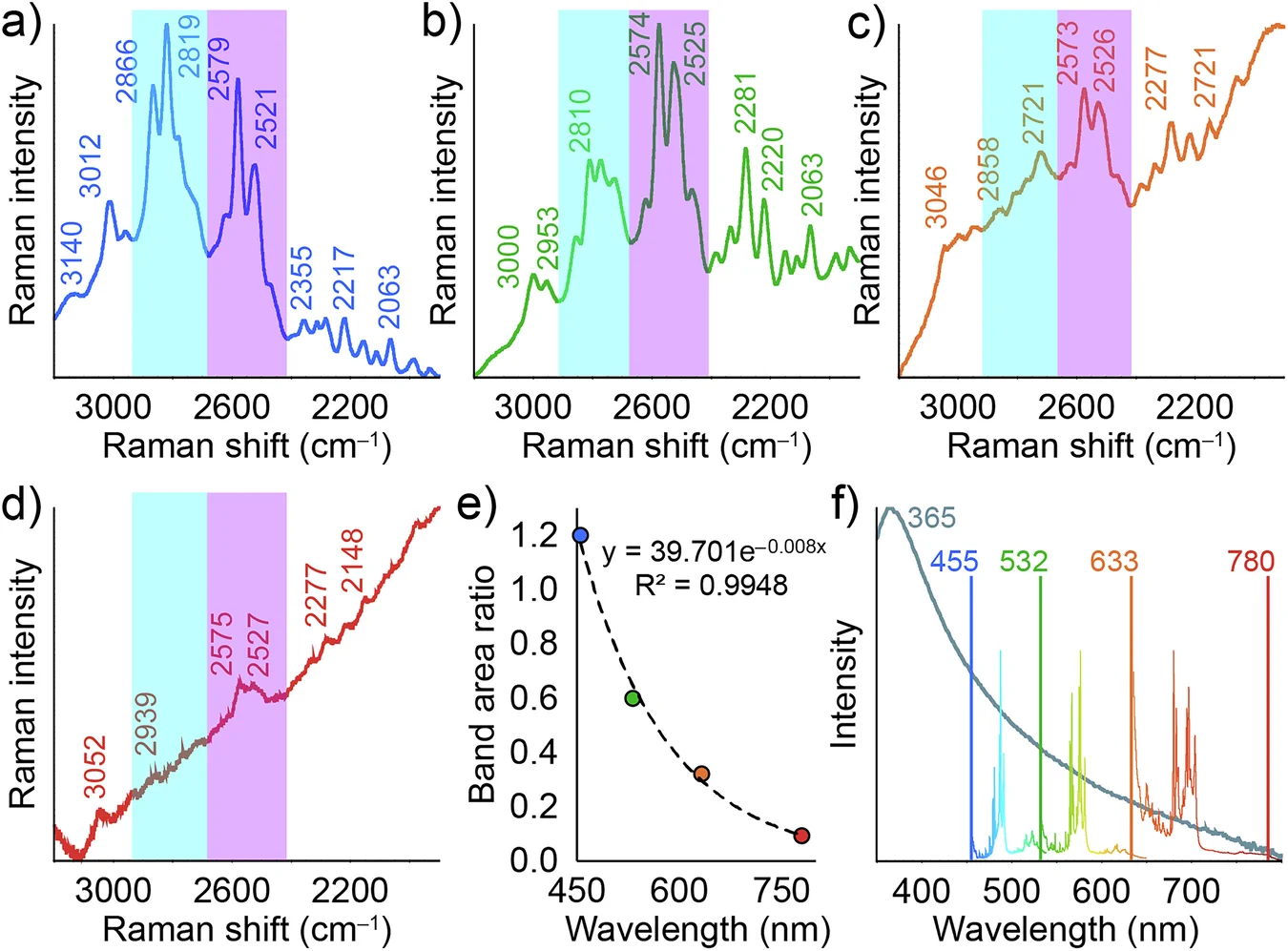
Excitation-wavelength dependence of high-order SERS transitions. Ag-SERS spectra of 4-ABT/4,4′-DMAB measured with excitation wavelengths of **a** 455, **b** 532, **c** 633, and **d** 780 nm. **e** Ratios of band areas calculated for the two highlighted regions for each excitation: **a** 2926.766–2677.030 cm⁻¹ (azure) and 2677.030–2410.901 cm⁻¹ (purple); **b** 2910.766–2670.580 cm⁻¹ (azure) and 2670.580–2409.272 cm⁻¹ (purple); **c** 2910.518–2667.531 cm⁻¹ (azure) and 2667.531–2412.974 cm⁻¹ (purple); **d** 2910.438–2667.451 cm⁻¹ (azure) and 2667.451–2476.533 cm⁻¹ (purple). **f** Schematic representation of individual excitation lines (marked), wavelengths of scattered Raman photons, and surface-plasmon extinction spectra of the Ag substrate. Raman spectra are displayed on a full-scale basis. The extinction spectrum of the Ag substrate was taken from the ref. 12.

Subsequently, we investigated the excitation wavelength dependence of the high-order transition region. Figure 3 shows that this effect is clearly observable when using different excitation wavelengths across the visible range. Not only are the relative band areas excitation-dependent, but the number of observable bands also decreases with increasing excitation wavelength. In the 532-nm excited spectrum (Fig. 3b), the two most intense bands are located at 2574 and 2525 cm⁻¹; however, several additional bands can also be identified around 3000, 2810, 2281, 2220, and 2063 cm⁻¹. Compared with the 455-nm case (Fig. 3a), the 532-nm spectrum shows lower relative intensities for bands above ~2600 cm⁻¹ and higher relative intensities for those below 2500 cm⁻¹. These differences, to some extent mirror those between the fundamentals excited at 455 nm (Fig. 1) and at 532 nm (Fig. S3). For example, the pair of bands at 1432 and 1389 cm⁻¹ is almost as intense as the 1142 and 1076 cm⁻¹ bands in the latter case. Similar relative intensity changes can also be observed at 633 nm (Fig. 3c), where the region is dominated by the bands at 2573 and 2526 cm⁻¹, while the intensities of the other bands remain approximately the same, though the overall intensity below 2400 cm⁻¹ is relatively reduced. This is consistent with the fundamentals excited at 633 nm (Fig. S4), where the overall intensity in the 1600–1000 cm⁻¹ region is similar and about two-thirds higher than in the region below 1000 cm⁻¹. At 780 nm excitation (Fig. 3d), the two most intense bands in the high-order region appear at 2575 and 2527 cm⁻¹, while the others are weak, though still detectable. In the corresponding fundamentals (Fig. S5), the most intense band is observed at 1079 cm⁻¹, with the remaining bands at least two times weaker. An interesting feature here is the relatively high intensity of the 388 cm⁻¹ band, which was not as pronounced in the previous cases, although a weaker effect is visible at 633 nm. While this band is unlikely to contribute directly to the high-order transitions in the investigated region, it may help explain the appearance of new maxima or band deformations at ~1490/1470 cm⁻¹ through possible combination with the intense 1079 cm⁻¹ band. The approximately constructed spectra for the excitation wavelengths of 532, 633, and 780 nm are shown in Fig. S6. However, due to differences in relative ratios caused by the effects discussed above, as well as lower overall intensity, it was not possible to fully reconstruct the exact spectra. Nevertheless, even when using all bands for construction, the positions of the spectral lines show good agreement with the experimental data.

The variability of relative band areas is quantified in Fig. 3e, which shows the dependence of area ratios between the regions 2910–2670 cm⁻¹ (azure rectangles) and 2670–2400 cm⁻¹ (purple rectangles), as displayed in Fig. 3a–d. The ratios decrease with increasing excitation wavelength, and the dependence follows an exponential trend. This behavior is consistent with previously reported excitation wavelength dependencies, where the relative intensities of 4,4´-DMAB bands compared to 4-ABT bands also decrease with increasing wavelength^12^. This suggests that the observed high-order transitions may directly reflect the number of dimer molecules on the surface. This interpretation is supported by our vibrational assignment, which shows that all recorded bands can be attributed to 4,4´-DMAB.

To further confirm that the changes in the relative intensities of high-order transition bands are associated with the ongoing photochemical reaction, additional measurements were performed using the same Ag-enhancing substrate and benzenethiol (BT), a molecule not expected to undergo photochemical transformation under the applied conditions. The results, obtained using excitation wavelengths of 532 nm, 633 nm, and 785 nm (Figs. S7–S9), show that the relative intensities of individual fundamental bands do not significantly vary with excitation wavelength, supporting the assumption that no photochemical transformation occurs in this case.

Furthermore, the BT spectra recorded at 532 nm and 633 nm clearly exhibit high-order transitions, while these features are only weakly visible at 785 nm. To confirm their origin, the same reconstruction approach as described for 4-ABT was applied. In this case, all fundamental vibrational modes were included in the construction of the high-order spectrum, as no selection based on photochemical transformation is required. The resulting reconstructed spectrum shows good agreement with the experimental data, confirming that the observed features can indeed be assigned to high-order transitions (Fig. S10). In addition, measurements performed at varying laser powers (633 nm excitation, Figs. S11, 12) show that the relative intensities of both fundamental and high-order transitions remain unchanged. This indicates that both sets of bands originate from the same molecular species and further supports the assignment of the observed features to high-order transitions. Importantly, the relative peak area ratios of the BT high-order bands remain nearly constant across the different excitation wavelengths. This behavior contrasts with the 4-ABT/4,4′-DMAB system and further supports the conclusion that the observed changes in relative intensities of high-order bands in that case are primarily driven by photochemical transformation.

In addition, minor variations in band area ratios may also arise from the spectral positions of the inelastically scattered photons relative to the maxima of the surface plasmon resonance. This is illustrated in Fig. 3f, where the Raman spectra are plotted against wavelength. The photons associated with high-order transitions are clearly located further from the excitation lines and thus farther from the plasmon resonance maximum than is typical for fundamental transitions. Specifically, for 532- and 633-nm excitation the transitions remain within the plasmon band region (though on its descending side), while for 780 nm they fall in a region where the plasmon band is nearly flat. This may also to some extent explain the decreasing intensity of high-order transitions.

Interestingly, high-order transitions in SERS have previously been reported mainly for resonant structures. This behavior can be further illustrated using methylene blue (MB), which typically interacts with plasmonic surfaces via non-covalent interactions involving its aromatic system and exhibits a strong electronic transition around 620 nm (Fig. S13)^27^. This is consistent with MB spectra measured on our silver substrate, where high-order transitions are most pronounced for excitation wavelengths of 532 and 633 nm (Figs. S14, 15), i.e., under conditions where the excitation significantly overlaps with the molecular absorption band. In contrast, for 785 nm excitation, which overlaps only weakly or not at all with this transition, the high-order features are considerably less intense (Fig. S16). In our case, however, the calculated absorption spectrum of 4-ABT shows its most intense band at 229 nm, which is too far from any of the applied excitation wavelengths. Nevertheless, 4-ABT contains a thiol group with a strong affinity to plasmonic surfaces, favoring the formation of covalently bound surface complexes. This likely induces a red shift of the HOMO–LUMO transition into the visible range, as demonstrated in our previous study and shown in Fig. S17^12^. Importantly, such a shift is likely to occur not only for 4-ABT, but also for its potential photochemical product, 4,4′-DMAB (Fig. S18), as well as for BT (Fig. S19), as they all possess a thiol group. This indicates that the maxima of the absorption bands of these surface complexes are shifted to the region between 500 and 600 nm, resulting in overlap with the excitation wavelengths used and thereby explaining the presence of high-order transitions. Crucially, the ability to systematically observe these high-order transitions under nominally non-resonant conditions for molecules that do not possess an electronic transition in resonance with the excitation wavelength in their native state highlights their importance as robust markers of covalent surface complex formation. Therefore, we propose that the observation and reconstruction of high-order transitions can serve as a powerful tool for probing the interaction between molecules and plasmonic substrates, providing valuable insight for investigating previously uncharacterized molecular–surface combinations. Importantly, until now, evidence for such covalent surface complexes has been largely limited to DFT calculations, underscoring the significance of our experimental observations.

### Substrate dependence

**Fig. 4:**
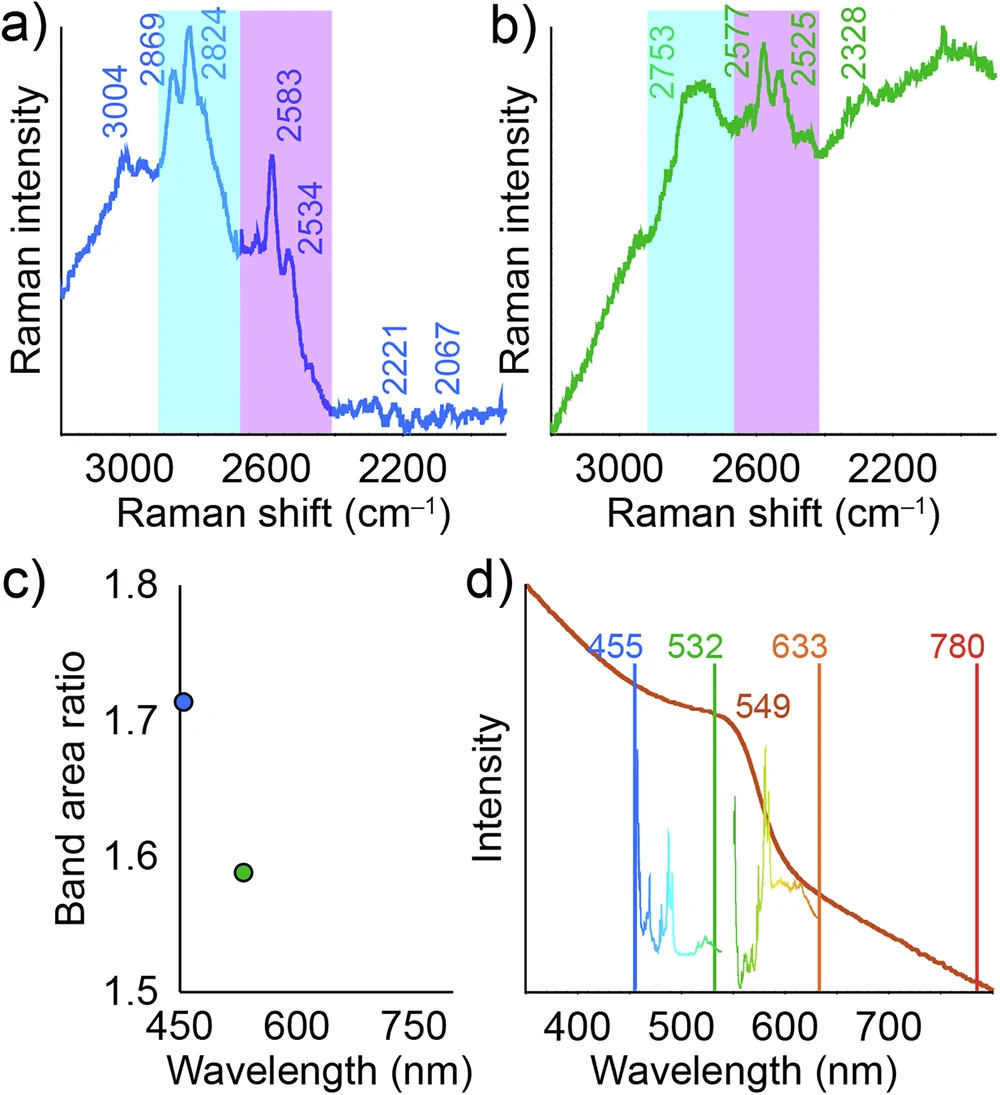
High-order SERS transitions on copper substrates. Cu-SERS spectra of 4-ABT/4,4′-DMAB measured with excitation wavelengths of **a** 455, and **b** 532 nm. **c** Ratios of band areas calculated for the two highlighted regions for each excitation: **a** 2926.766–2677.030 cm⁻¹ (azure) and 2677.030–2410.901 cm⁻¹ (purple); **b** 2910.766–2670.580 cm⁻¹ (azure) and 2670.580–2409.272 cm⁻¹ (purple). **d** Schematic representation of individual excitation lines (marked), wavelengths of scattered Raman photons, and surface-plasmon extinction spectra of the Cu substrate. Raman spectra are displayed on a full-scale basis. The extinction spectrum of the Cu substrate was taken from the ref. 12.

To extend the validity of the presented findings, we have performed an equivalent set of experiments using gold- and copper-based enhancing substrates. Measurements performed on an electrodeposited Au substrate using multiple excitation wavelengths (Figs. S20–S22) did not reveal observable overtone or combination bands under the studied conditions, although weak fundamental modes are present in the corresponding spectral region. This may be related to a lower extent of the photon-induced transformation on this substrate, resulting in weaker fundamental modes of the dimer species and, consequently, in high-order transitions of insufficient intensity to be detected. These observations also indicate that the fundamental modes around 3000 cm⁻¹ do not significantly distort the appearance of high-order bands in the case of silver, as their intensity is considerably lower than that of the high-order bands. Interestingly, high-order transitions were observed on the copper substrate. Although this system was investigated using all the same excitation wavelengths as in the case of silver, only the two lowest wavelengths, i.e., 455 and 532 nm, proved to be SERS-active in this case, while SERS spectra for these two excitation wavelengths are shown in Figs. S23, 24. Briefly, both spectra exhibited high intensity of 4,4´-DMAB bands, similarly to those observed in the case of silver. While the situation for copper is more complicated because of the bands attributed to the copper oxides, it has no direct impact on the bands in the high-order transition area, as those bands are located below 650 cm⁻¹, thus being out of reach for investigated area by combination with any other fundamental bands (and at the same time ruled out because of the nature of high-order transitions, which cannot happen across two different molecules). High-order transitions recorded on a copper substrate are shown in Fig. 4. For the case of excitation wavelength of 455 nm (Fig. 4a**)**), there are several bands well observable, namely at 3004, 2869, 2824, 2583, and 2534 cm⁻¹, which is in good agreement with the transitions recorded for the silver substrate using the same laser wavelength (Fig. 3a). Similarly, the relative ratios are similar, meaning that the area of bands higher than 2600 cm⁻¹ is more intense than the area below this value. For the second SERS-active excitation wavelength, there is still a group of observable bands, although their number is lower and they are not as intense as in the case of silver (Fig. 4b). Well distinguishable bands for this wavelength are located at 2753, 2577, and 2525 cm⁻¹, although the first named is clearly composed of several components. Nevertheless, the excitation dependence is similar to that observed in the case of the silver substrate, meaning that the ratio between the named areas is lower than in the case of the first used excitation wavelength (Fig. 4c), although, because of the low number of points caused by the SERS-inactivity of other used excitation wavelengths, no more can be stated about the nature of this dependence. One of the reasons for the lower SERS activity may be the generally lower enhancing ability of copper, which is well-known to the community. Therefore, although its plasmon bands are located more suitably in relevance of used excitation wavelengths and Raman photons (Fig. 4d), the generally lower intensity may be the real reason why no SERS was observed for the case of the two highest excitation wavelengths (meaning that, thanks to the position of plasmon band, it is possible at least to obtained SERS signal from the lower two wavelengths). Nevertheless, the fact that the trend is similar to the one observed for the silver and that the excitation wavelengths located in the close vicinity of the maxima of the suggested molecule–metal surface complexes support the findings presented so far, while indicating the feasibility of the high-order transition investigation in a general manner, more than the specific case for silver.

### Generality of the concept

Importantly, all these observations further suggest that, while electromagnetic enhancement is necessary to achieve sufficient signal intensity, it is not sufficient to explain the appearance of high-order transitions. Instead, chemical enhancement mechanisms, particularly those involving resonance with electronic transitions of the molecule or molecule–surface complex, appear to play a decisive role. Under such conditions, the presence of resonance governs both the visibility and the relative intensity of high-order bands. The ongoing chemical transformations are manifested through the dependence of the high-order bands’ relative intensities on the excitation wavelength used, reflecting different reaction kinetics under different excitation wavelengths. This is further confirmed by additional measurements performed on acetaminophen (AAP), which is also capable of forming photoactive covalently bound surface complexes (Supplementary Note 1). Furthermore, the fact that the 4-ABT/4,4′-DMAB spectra were measured using a different Raman microscope than BT, MB, and AAP (see Methods section), rules out the possibility that the observed findings are the result of experimental error.

## Conclusion

In this work, we demonstrate a systematic reconstruction and assignment of high-order Raman transitions of 4-ABT/4,4´-DMAB, revealing them as a direct and robust marker of molecule–metal surface complex formation. By combining theoretical spectra constructed from selected fundamentals with experimental measurements, we show that all observed high-order features arise exclusively from 4,4´-DMAB bands, with anharmonicity accounting for minor deviations.

The excitation-wavelength dependence consistently reveals a decrease in the relative intensity of the high-order bands with increasing wavelength, a trend reproducible on both silver and copper substrates, further confirming the direct link between the high-order transition profile and ongoing chemical processes in the system. By employing different plasmonic metals and model probes, we demonstrate that, although electromagnetic enhancement is required to achieve sufficient signal intensity, the observed high-order bands and their appearance are primarily governed by chemical enhancement mechanisms, particularly those involving resonance with electronic transitions of the molecule or molecule–surface complex, whether present in the native state or induced by covalent interaction with the plasmonic surface.

These findings establish high-order Raman transitions as a reliable signature of surface complex formation and indicate their potential broader applicability for probing molecule–metal interactions with high sensitivity. The results provide insight into SERS mechanisms and highlight the value of analyzing high-order transitions to study surface-confined reactions in other systems.

## Methods

### Materials

The study employed the following chemicals: 4-aminobenzenethiol (Fluka, 95%), acetaminophen (Sigma-Aldrich, 98%), benzenethiol (Sigma-Aldrich, ≥98%), methylene blue (Sigma-Aldrich, ≥82%), ammonia solution (Lachner, 26%), silver nitrate (Sigma-Aldrich, 99.99%), sodium hydroxide (Penta, 98%), ammonium chloride (Penta, 99.5%), copper(II) chloride (Sigma-Aldrich, 97%), gold(III) chloride (Sigma-Aldrich, 99.99%), sulfuric acid (Lachner, 96%), methanol (Lachner, 99.95%), aluminum oxide (Sigma-Aldrich, 99.5%), hydrogen peroxide (Penta, 30%), and calcium carbonate (Lachner, 99%). All reagents were used as received from the suppliers without further purification. Milli-Q water (Millipore system) was employed for the preparation of all aqueous solutions. For the deposition baths, 4-ABT and BT were dissolved in methanol, whereas AAP and MB were dissolved in water.

### Nanostructured substrate preparation

Large-area Ag, Au, and Cu SERS substrates were fabricated by electrodeposition onto platinum targets^12,28,29^. Before deposition, the Pt targets were polished with metallographic papers, followed by polishing with Al₂O₃ and CaCO₃, and subsequently cleaned by immersion in a H₂SO₄/H₂O₂ mixture (3:1 v/v) for 30 min. The substrates were then rinsed thoroughly with Milli-Q water and dried. Electrochemical deposition was performed using specific current sequences in the respective electrochemical baths (Table S2).

The resulting Ag and Au substrates exhibited dendritic surface structures, while the Cu substrates displayed characteristic spherical, “cauliflower-like” morphologies. A detailed structural characterization of these substrates has been previously reported^12,28,29^.

For molecular functionalization, the prepared substrates were immersed in an analyte deposition bath (base concentration 1 × 10⁻⁴ mol·dm⁻³) for 24 h. After incubation, the samples were removed, rinsed with methanol, dried under a nitrogen stream, and stored in PE sample boxes without further treatment until use.

### SERS spectroscopy

A DXR Raman microscope (Thermo Scientific Nicolet, USA) was employed for recording SERS spectra of 4-ABT. Excitation wavelengths in the visible region (455, 532, and 633 nm) and in the near-infrared region (780 nm) were employed for these experiments. The system is equipped with four interchangeable laser sources: diode lasers (455 and 780 nm), a diode-pumped Nd: YAG laser (532 nm), and a He–Ne gas laser (633 nm). The scattered light was detected using a multichannel charge-coupled device (CCD) detector. All spectra, regardless of the excitation wavelength, were acquired with an instrumental spectral resolution of 4.4 cm⁻¹, using a laser power set to 1 mW and an integration time of 20 × 1 s.

For additional measurements of acetaminophen (AAP), benzenethiol (BT), and methylene blue (MB), an inVia Raman microscope (Renishaw, UK) equipped with excitation wavelengths of 532 nm (Nd: YAG) and 633 nm (He–Ne) was used. All spectra were acquired using a 100× objective (NA 0.85), while the laser power, integration time, and number of accumulations for each excitation wavelength and investigated molecule are summarized in Table S3. All measurements were performed with a fixed instrumental spectral resolution of 2 cm⁻¹, and the signal was acquired using a thermoelectrically cooled CCD detector.

### DFT calculations

All DFT calculations were performed using the B3LYP functional and the LanL2DZ basis set as implemented in Gaussian 16 W software. First, geometry optimization was carried out for each molecule in its isolated (gas-phase) state. Subsequently, a silver atom was attached to the most probable binding functional group, and the resulting metal–molecule complexes were re-optimized using the same level of theory. Finally, UV/Vis absorption spectra were computed for both the native molecules and their silver-complexed forms using time-dependent DFT (TD-DFT) at the same functional and basis set level.

### Supporting information

Raman spectrum of crystalline 4-ABT; high-order transition reconstruction procedure; SERS spectra of 4-ABT/4,4′-DMAB recorded at excitation wavelengths of 532, 633, and 780 nm; assignment of fundamental vibrational modes of 4,4′-DMAB; description of used electrochemical baths and current sequences for platinum plate targets; reconstruction of high-order transitions for 4-ABT/4,4′-DMAB spectra excited at 532, 633, and 780 nm; SERS spectra of benzenethiol (BT); reconstruction of high-order transitions for BT spectra; power dependence of BT SERS spectra; SERS spectra of methylene blue (MB); SERS spectra of acetaminophen (AAP); calculated UV/Vis absorption spectra; experimental parameters for Raman measurements of AAP, BT, MB.

## Supplementary information

Transparent Peer Review file

Supporting Information

## Data Availability

The datasets generated and analyzed during the current study are available in the Zenodo repository at https://doi.org/10.5281/zenodo.20531921.
