# Supplementary material for: Reconstructing high-order SERS transitions in an overlooked spectral region reveals surface complex formation
Source: Commun Chem. 2026 Jul 17;9:353. doi: 10.1038/s42004-026-02136-x (PMC13748603; doi:10.1038/s42004-026-02136-x)
Supplement: Supplementary file 1 [file 42004_2026_2136_MOESM1_ESM.pdf]

nature portfolio

Peer Review File

Reconstructing High-Order SERS Transitions in an Overlooked Spectral Region Reveals Surface Complex Formation

Corresponding Author: Dr Ivan Kopal

This file contains all reviewer reports in order by version, followed by all author rebuttals in order by version.

Version 0:

Reviewer comments:

Reviewer #1

(Remarks to the Author)
Dear editor
Please find here my review about the article “Underestimated Region: Reconstructing High-Order SERS Transitions to Reveal Surface-Complex Formation”.
This article describes SERS measurements of aminobenzenethiol molecules, with a specific focus on the high wavenumbers region (beyond 2000 cm-1). The idea is interesting and original, yet I have a few comments and questions:
-The authors choose silver or copper as SERS substrates but those two metals are known to be reactive in the air. Why haven’t they try with gold ? This metal is highly SERS efficient and much more stable. This would avoid the question of the role of metal oxides in the SERS spectra.
-The authors choose 4-ABT As SERS molecule, which is well known to be reactive (formation of DMAB) during SERS measurements. It would be interesting to compare with a similar but les reactive molecule, like benzenethiol. According to the results presented in the article, no high orders modes should be observed as they originated from the formation of DMAB, that cannot be formed from benzenethiol.
-For the analysis of the 3000 cm-1 region the authors have not considered the presence of CH stretching modes. Why? I suggest to add the Raman spectrum of bulk ABT for the sake of comparison. For instance, in the Raman spectrum of benzene (from https://sisu.ut.ee/heritage-analysis/32-raman-spectroscopy/) highly intense CH stretching modes are present in the 3000 cm-1 region. Why should they not be present in the SERS spectrum ?
-I recommend to look at the TERS (Tip Enhanced Raman Spectroscopy) bibliography on the same molecule.
-It is not usual to plot SERS spectra with the highest wavenumber region on the left side of the horizontal axis. This is typically done for IR, but not for Raman.
-For the interpretation of the high wavenumbers’ region, the authors have used only modes from DMAB in the calculations of the shift. Why? Once again it should be interesting to make the comparison with benzenethiol.
-In SERS measurement, the formation of DMAB from ABT is observed only if the laser power is rather high. I recommend the authors making SERS measurements using very low laser power. This would avoid the formation of DMAB and confirm if this molecule is essential for the interpretation of the 3000 cm-1 regions.
-The SERS results with copper are inconclusive, once again I recommend using gold instead.
-The measurements with acetaminophen are just preliminary results and do not bring any clear information. The authors should put forward their analysis or remove this part.
As a conclusion, the authors propose an original and promising approach for the interpretation of the 3000 cm-1 region in SERS spectra. Nevertheless, complementary measurements should be performed (SERS with gold nanostructures, low laser power measurements, SERS with benzenethiol, bulk Raman spectrum of ABT) and added in the discussion part to support their hypothesis.

Reviewer #2

(Remarks to the Author)
This study investigates the origin of high-order bands (overtones and combination bands) in surface-enhanced Raman scattering (SERS) spectroscopy, proposing these bands as markers for surface-complex formation, with experimental

validation using 4-aminobenzenethiol (4-ABT) as a model molecule. The topic demonstrates a degree of novelty, particularly in linking high-order band analysis with surface photochemical reactions, which has the potential to significantly enhance the interpretation of SERS spectra and the research on interfacial processes. However, the article presents notable shortcomings in the rigor of its data logic and the depth of its explanations. Consequently, I cannot currently recommend this manuscript for publication in Communications Chemistry. The following specific comments are provided for the authors' reference.

1. The authors consider that the high-order SERS bands originate entirely from surface-formed 4,4'-DMAB and can serve as markers for surface complexes. However, the current data do not yet fully prove a direct correlation between the appearance of high-order bands and surface complexes. Is it possible that on substrates with high enhancement factors, any strongly adsorbed molecule with suitable symmetry could produce observable high-order bands? The authors are encouraged to incorporate control experiments. For instance, selecting a molecule known to adsorb onto the Ag surface through physical interactions (e.g., π-π stacking, electrostatic forces) rather than chemical bonding, and testing under identical experimental conditions (particularly the same substrate and excitation wavelength) would help determine if similar high-order bands emerge. Alternatively, employing a thiol molecule recognized for its lack of surface photochemical reactions for comparison would be beneficial.
2. Why does the relative intensity ratio of the high-order bands (Figure 3e) vary with wavelength? Is this variation directly attributable a resonance Raman effect of the DMAB molecule, or dose it relates to charge-transfer resonance with the metal?
3. The authors mention that the surface complex may induce a red-shift in the HOMO-LUMO gap but fail to provide experimental (e.g., UV-Vis) or computational evidence to support the existence of an electronic resonance for this complex at the employed excitation wavelengths.
4. The authors must explore the underlying physical mechanism responsible for the enhancement of high-order bands more thoroughly. Is it merely a byproduct of pure electromagnetic field enhancement, or is it specifically linked to chemical enhancement (charge transfer)?
5. It is recommended that the authors apply their current methodology to a novel, controversial molecule-metal system to demonstrate that this approach can effectively address interfacial chemistry problems that are difficult to elucidate using existing techniques, such as conventional SERS or DFT. Otherwise, the manuscript may be better suited for submission to a specialized journal focused on spectroscopic characterization.

Version 1:

Reviewer comments:

Reviewer #1

(Remarks to the Author)
The authors carried out the suggested additional measurements and responded thoroughly to my comments. I am somewhat surprised that DMAB modes were not observed in the gold samples, but their conclusions remain consistent.
I therefore recommend the publication of this article.

Reviewer #2

(Remarks to the Author)
The authors have revised the manuscript and adequately addressed all reviewer comments. The quality of the manuscript is now satisfactory. Thus, I recommend the publication of this manuscript in Communications Chemistry.

Open Access This Peer Review File is licensed under a Creative Commons Attribution 4.0 International License, which permits use, sharing, adaptation, distribution and reproduction in any medium or format, as long as you give appropriate credit to the original author(s) and the source, provide a link to the Creative Commons license, and indicate if changes were made.
In cases where reviewers are anonymous, credit should be given to 'Anonymous Referee' and the source.
The images or other third party material in this Peer Review File are included in the article's Creative Commons license, unless indicated otherwise in a credit line to the material. If material is not included in the article's Creative Commons license and your intended use is not permitted by statutory regulation or exceeds the permitted use, you will need to obtain permission directly from the copyright holder.
To view a copy of this license, visit https://creativecommons.org/licenses/by/4.0/

Reviewer #1 (Remarks to the Author):

Dear editor

Please find here my review about the article "Underestimated Region: Reconstructing High-Order SERS Transitions to Reveal Surface-Complex Formation". This article describes SERS measurements of aminobenzenethiol molecules, with a specific focus on the high wavenumbers region (beyond 2000 cm-1). The idea is interesting and original, yet I have a few comments and questions:

-The authors choose silver or copper as SERS substrates but those two metals are known to be reactive in the air. Why haven't they try with gold ? This metal is highly SERS efficient and much more stable. This would avoid the question of the role of metal oxides in the SERS spectra.

We thank the reviewer for this insightful suggestion. We agree that gold represents a stable and widely used SERS substrate and provides an important point of comparison. In response, we have included additional measurements performed on a gold substrate prepared by electrodeposition to ensure consistency with the silver and copper substrates used in this work. The corresponding spectra, measured using three different excitation wavelengths, have been added to the Supporting Information (Figures S20–S22).

In contrast to Ag and Cu, no overtone or combination bands were observed for the Au substrate under the studied conditions, although weak fundamental vibrational features in the relevant spectral region are still present. This observation is consistent across all excitation wavelengths employed. This behavior may be related to the lower intensity of the fundamental modes associated with the 4,4′-DMAB species on Au, which would consequently lead to even weaker higher-order transitions. However, a detailed investigation of substrate-dependent reaction pathways and optimization of SERS activity is beyond the scope of the present work. We have added a brief discussion of this point to the manuscript:

*"Measurements performed on an electrodeposited Au substrate using multiple excitation wavelengths (**Figures S20-S22**) did not reveal observable overtone or combination bands under the studied conditions, although weak fundamental modes are present in the corresponding spectral region. This may be related to a lower extent of the photon-induced transformation on this*

substrate, resulting in weaker fundamental modes of the dimer species and, consequently, in high-order transitions of insufficient intensity to be detected."

-The authors choose 4-ABT as SERS molecule, which is well known to be reactive (formation of DMAB) during SERS measurements. It would be interesting to compare with a similar but less reactive molecule, like benzenethiol. According to the results presented in the article, no high orders modes should be observed as they originated from the formation of DMAB, that cannot be formed from benzenethiol.

We would like to thank the reviewer for this important suggestion. First, we would like to clarify that high-order transitions do not necessarily arise exclusively from DMAB, but may, in general, originate from any molecule or molecule–surface complex possessing an electronic transition that overlaps with the excitation wavelength and/or the inelastically scattered photons. Therefore, in principle, 4-ABT itself could also exhibit high-order transitions; however, in our case, these are not of sufficient intensity to be experimentally observed.

The fact that the observed high-order transitions are associated with the formation of DMAB in our system is further supported by measurements performed on a Au substrate. In this case, the relative intensities of DMAB-related bands are significantly lower compared to Ag and Cu, and no high-order transitions were observed, with only weak fundamental aromatic vibrations present in the ~3000 cm^{-1} region.

Nevertheless, we agree that comparison with a less reactive molecule provides valuable complementary information. Therefore, we have performed additional measurements using benzenethiol adsorbed on an Ag substrate under conditions closely matching those used for 4-ABT, including three different excitation wavelengths. The obtained spectra are very similar across all excitation wavelengths, indicating that the molecular structure remains unchanged under the applied experimental conditions.

Furthermore, high-order transitions are observed in the case of benzenethiol, particularly for excitation wavelengths of 532 and 633 nm, and to a lesser extent also at 785 nm. Their relative intensities do not vary significantly with excitation wavelength, in contrast to the behavior observed for the 4-ABT/DMAB system. This supports the conclusion that the variability of high-order transitions in the 4-ABT case is linked to ongoing photochemical transformation.

The presence of high-order transitions in benzenethiol is likely related to the relatively high intensity of its fundamental vibrational modes. This may be associated with the formation of a molecule–surface complex of a similar nature to that of 4-ABT, as both molecules contain a thiol group and are known to form self-assembled monolayers on metal surfaces. To further support this assumption, we have calculated the UV–Vis spectrum of benzenethiol covalently bound to silver using the same computational approach as for 4-ABT. The results show that the absorption band of the formed complex is shifted to the 500–600 nm region (Figure S19), which corresponds well to the excitation wavelengths where the high-order transitions are most pronounced.

Finally, we attempted to reconstruct the high-order bands using the approach described in the present work. For this purpose, all fundamental vibrational modes of benzenethiol in the 1800–600 cm^{-1} region were considered. The reconstruction was performed by shifting the experimental spectrum by the wavenumber values corresponding to individual vibrational modes and subsequently summing these shifted spectra with weights proportional to the relative intensities of the respective fundamental bands. The result (Figure S10) shows a reasonable agreement with the experimental spectra, although some bands are partially overlapped or slightly shifted, which is a commonly observed feature in the high-order transition region.

The corresponding discussion has been added to the manuscript:

"*To further confirm that the changes in the relative intensities of high-order transition bands are associated with the ongoing photochemical reaction, additional measurements were performed using the same Ag-enhancing substrate and benzenethiol (BT), a molecule not expected to undergo photochemical transformation under the applied conditions. The results, obtained using excitation wavelengths of 532 nm, 633 nm, and 785 nm (Figures S7–S9), show that the relative intensities of individual fundamental bands do not significantly vary with excitation wavelength, supporting the assumption that no photochemical transformation occurs in this case.*

Furthermore, the BT spectra recorded at 532 nm and 633 nm clearly exhibit high-order transitions, while these features are only weakly visible at 785 nm. To confirm their origin, the same reconstruction approach as described for 4-ABT was applied. In this case, all fundamental vibrational modes were included in the construction of the high-order spectrum, as no selection based on photochemical transformation is required. The resulting reconstructed spectrum shows a good agreement with the experimental data, confirming that the observed features can indeed be

assigned to high-order transitions (Figure S10). In addition, measurements performed at varying laser powers (633-nm excitation, Figures S11-S12) show that the relative intensities of both fundamental and high-order transitions remain unchanged. This indicates that both sets of bands originate from the same molecular species and further supports the assignment of the observed features to high-order transitions. Importantly, the relative peak area ratios of the BT high-order bands remain nearly constant across the different excitation wavelengths. This behavior contrasts with the 4-ABT/4,4'-DMAB system and further supports the conclusion that the observed changes in relative intensities of high-order bands in that case are primarily driven by photochemical transformation."

In addition, we have carefully revised the manuscript to ensure that the discussion does not imply that high-order transitions in SERS are exclusively associated with the formation of DMAB, and that this distinction is clearly communicated to the reader.

-For the analysis of the 3000 cm-1 region the authors have not considered the presence of CH stretching modes. Why? I suggest to add the Raman spectrum of bulk ABT for the sake of comparison. For instance, in the Raman spectrum of benzene (from https://sisu.ut.ee/heritage-analysis/32-raman-spectroscopy/) highly intense CH stretching modes are present in the 3000 cm-1 region. Why should they not be present in the SERS spectrum ?

We would like to thank the reviewer for this valuable suggestion. We agree that in conventional vibrational spectroscopy aromatic molecules typically exhibit intense CH stretching modes in the ~3000 cm^{-1} region.

However, in SERS, the intensity of these vibrations can be significantly reduced due to several factors, such as their distance from the metal surface, unfavorable molecular orientation, and generally weaker enhancement, as discussed in the Introduction. While CH stretching modes may still be observed in certain cases, their intensity is often limited by surface selection rules.

Therefore, it cannot be excluded that weak contributions from CH stretching fundamentals may overlap with the high-order transitions in the ~3000 cm^{-1} region. However, such contributions would be expected to be minor and likely manifest only as a slight increase in the spectral background rather than as distinct bands. In addition, CH stretching fundamentals are not expected to contribute in the 2800–1900 cm^{-1} region, where high-order transitions are also observed.

This interpretation is further supported by measurements performed on the Au substrate. In this case, where high-order transitions are not observed, only very weak CH stretching fundamentals are detectable, indicating that these modes have insufficient intensity to significantly influence the spectral appearance in this region.

Nevertheless, we agree that this point should be clarified in the manuscript, and the corresponding discussion has been added to the Results section:

"*It should be noted that although aromatic molecules typically exhibit distinctive –CH stretching modes in the ~3000 cm⁻¹ region in conventional Raman spectroscopy, these vibrations are often weak in SERS due to their distance from the metal surface, unfavorable orientation, and lower enhancement efficiency. Therefore, any contribution of –CH stretching fundamentals to the spectra presented here is expected to be minor and may only manifest as a slight background in the high-order transition region.*"

"*These observations also indicate that the fundamental modes around 3000 cm⁻¹ do not significantly distort the appearance of high-order bands in the case of silver, as their intensity is considerably lower than that of the high-order bands.*"

Furthermore, we agree that including the spectrum of bulk 4-ABT provides a useful reference. Therefore, we have added the Raman spectrum of crystalline 4-ABT (measured using 532 nm excitation) to the Supporting Information (Figure S1). This spectrum does not exhibit bands attributable to 4,4′-DMAB and does not show features in the region where high-order transitions are observed in the SERS spectra. In addition, the relative intensity of CH stretching modes around 3000 cm⁻¹ is higher compared to the SERS spectra, further supporting their reduced enhancement under SERS conditions.

-I recommend to look at the TERS (Tip Enhanced Raman Spectroscopy) bibliography on the same molecule.

We thank the reviewer for this valuable suggestion. We agree that tip-enhanced Raman spectroscopy provides important insight into the behavior of 4-ABT at the nanoscale, particularly with respect to plasmon-induced transformations and the activation of additional vibrational modes. To address this point, we have briefly incorporated relevant TERS literature into the manuscript. Specifically, we now mention that TERS studies have demonstrated the coexistence

of 4-ABT and its dimerization product, as well as the appearance of additional vibrational features attributed to strong local field gradients. The following sentence has been added to the manuscript:

"*Furthermore, the photoreaction may not proceed in a straightforward manner, leading to the coexistence of multiple 4-ABT-derived species (4-ABTs) on the surface, as also supported by tip-enhanced Raman spectroscopy (TERS) studies, which revealed the presence of both 4-ABT and its dimerization product (DMAB), along with additional vibrational features attributed to field-gradient effects.*[24-26]"

We believe that this addition appropriately acknowledges the relevance of TERS studies while maintaining the focus and scope of the present work.

-It is not usual to plot SERS spectra with the highest wavenumber region on the left side of the horizontal axis. This is typically done for IR, but not for Raman.

We thank the reviewer for this observation. We are aware that the representation of the wavenumber axis may vary between infrared and Raman spectroscopy. In SERS spectroscopy, both conventions (increasing and decreasing wavenumber from left to right) can be found in the literature, and the choice is often guided by established practice within a given research group or by consistency across a particular study. In our case, we have followed the convention commonly used in our previous works and within our research group. For the sake of consistency throughout the manuscript, we have retained the current axis orientation. However, we would be happy to modify the axis direction if the reviewer or editor considers it necessary.

-For the interpretation of the high wavenumbers' region, the authors have used only modes from DMAB in the calculations of the shift. Why? Once again it should be interesting to make the comparison with benzenethiol.

We thank the reviewer for this comment. We would like to clarify that the reconstruction procedure was not restricted to modes of DMAB only. In the initial steps, all observable fundamental bands were considered for the generation of combination spectra, including those of 4-ABT. However, it was found that the best agreement with the experimental high-order region was achieved when using a subset of bands corresponding to DMAB. This reflects the fact that these modes exhibit the highest intensities in the experimental spectra and therefore contribute most significantly to the resulting high-order transitions. Consequently, only these bands were used for the final

interpretation of the high-order region. To avoid any ambiguity, we have revised the manuscript to make this point more explicit:

"*In the final step, we performed a weighted average using only selected "combination spectra." Although all fundamental bands were initially considered, the best agreement was achieved when using spectra shifted by 1438, 1390, and 1141 cm^{-1}, corresponding to the most intense modes of the DMAB species*."

In addition, as suggested by the reviewer, we have performed an analogous reconstruction for benzenethiol using the same approach. In this case, all observable fundamental bands were included without further selection, as benzenethiol does not undergo comparable photoinduced transformations under the studied conditions and therefore does not require separation into contributions from different species. The resulting spectrum has been added to the Supporting Information (Figure S10) and shows a reasonable agreement with the experimental high-order region, further supporting the general applicability of the proposed approach.

We have added a brief discussion of this comparison to the manuscript:

"*To confirm their origin, the same reconstruction approach as described for 4-ABT was applied. In this case, all fundamental vibrational modes were included in the construction of the high-order spectrum, as no selection based on photochemical transformation is required. The resulting reconstructed spectrum shows a good agreement with the experimental data, confirming that the observed features can indeed be assigned to high-order transitions (Figure S10).*"

-In SERS measurement, the formation of DMAB from ABT is observed only if the laser power is rather high. I recommend the authors making SERS measurements using very low laser power. This would avoid the formation of DMAB and confirm if this molecule is essential for the interpretation of the 3000 cm-1 regions.

We thank the reviewer for this valuable suggestion. We agree that reducing laser power can suppress the formation of DMAB and may provide additional insight into the origin of the observed spectral features. However, under very low laser power conditions, the overall SERS signal becomes too weak to reliably observe high-order transitions, making such measurements difficult to interpret in the context of the present study.

Instead, the role of DMAB in the appearance of high-order transitions is supported by the measurements performed on the Au substrate, where the formation of DMAB is significantly reduced and no high-order transitions are observed.

In addition, we have investigated the laser power dependence using benzenethiol as a non-reactive reference system. These measurements were performed at 633 nm excitation, where stable spectra with sufficient signal-to-noise ratio could be obtained across a range of laser powers. Importantly, when normalized, the spectra recorded at different laser powers show nearly identical relative intensities of both fundamental and high-order transitions. This indicates that these bands originate from the same molecular species and are not affected by power-induced transformations.

These observations further support the conclusion that, in the case of 4-ABT, the appearance of high-order transitions is closely related to photoinduced chemical processes rather than being an intrinsic feature observable under all conditions.

A brief discussion of this point has been added to the manuscript:

"*In addition, measurements performed at varying laser powers (633 nm excitation, Figures S11-S12) show that the relative intensities of both fundamental and high-order transitions remain unchanged. This indicates that both sets of bands originate from the same molecular species and further supports the assignment of the observed features to high-order transitions.*"

-The SERS results with copper are inconclusive, once again I recommend using gold instead.

We would like to thank the reviewer for this suggestion. As discussed above, measurements performed on gold substrates do not provide high-order transitions with sufficient intensity to allow for detailed mechanistic interpretation based on these features alone. Nevertheless, we believe that the comparison between Ag, Cu, and Au substrates provides important insight. In particular, the Ag- and Cu-based SERS spectra exhibit significantly more intense bands attributable to the DMAB species, whereas the relative intensities of these bands are substantially lower in the case of Au.

This trend correlates with the presence of high-order transitions, which are clearly observed for Ag and Cu substrates but are absent in the Au spectra. Taken together, these observations support the conclusion that the formation of DMAB is closely associated with the appearance of high-

order transition bands in the studied systems. For this reason, we believe that retaining the Cu-based measurements is important, as they provide complementary information to the Ag results. At the same time, we have extended this part of the manuscript by including a comparison with Au substrates to further support the interpretation, as mentioned in our previous answers.

-The measurements with acetaminophen are just preliminary results and do not bring any clear information. The authors should put forward their analysis or remove this part.

We thank the reviewer for this important comment. We agree that the acetaminophen measurements, in their original form, may appear preliminary and do not provide a sufficiently detailed analysis to support a full discussion within the main text. To address this point, we have revised the manuscript accordingly. The acetaminophen data have been moved to the Supporting Information, where they are presented as complementary results. These measurements were originally included to demonstrate that the observation of high-order transitions is not limited to the 4-ABT/DMAB system and to provide an additional control measured on a different experimental setup. However, we agree that benzenethiol now serves as a more systematic and better-controlled reference within the main text.

A brief mention of the acetaminophen results has been retained in the manuscript to highlight the broader applicability of the observed phenomena, while keeping the main discussion focused. We believe that this revision improves the clarity and focus of the manuscript.

"*This is further confirmed by additional measurements performed on acetaminophen (AAP), which is also capable of forming photoactive covalently bound surface complexes (Supplementary Note 1).*"

As a conclusion, the authors propose an original and promising approach for the interpretation of the 3000 cm-1 region in SERS spectra. Nevertheless, complementary measurements should be performed (SERS with gold nanostructures, low laser power measurements, SERS with benzenethiol, bulk Raman spectrum of ABT) and added in the discussion part to support their hypothesis.

Reviewer #2 (Remarks to the Author):

This study investigates the origin of high-order bands (overtones and combination bands) in surface-enhanced Raman scattering (SERS) spectroscopy, proposing these bands as markers for surface-complex formation, with experimental validation using 4-aminobenzenethiol (4-ABT) as a model molecule. The topic demonstrates a degree of novelty, particularly in linking high-order band analysis with surface photochemical reactions, which has the potential to significantly enhance the interpretation of SERS spectra and the research on interfacial processes. However, the article presents notable shortcomings in the rigor of its data logic and the depth of its explanations. Consequently, I cannot currently recommend this manuscript for publication in Communications Chemistry. The following specific comments are provided for the authors' reference.

1. The authors consider that the high-order SERS bands originate entirely from surface-formed 4,4'-DMAB and can serve as markers for surface complexes. However, the current data do not yet fully prove a direct correlation between the appearance of high-order bands and surface complexes. Is it possible that on substrates with high enhancement factors, any strongly adsorbed molecule with suitable symmetry could produce observable high-order bands? The authors are encouraged to incorporate control experiments. For instance, selecting a molecule known to adsorb onto the Ag surface through physical interactions (e.g., π-π stacking, electrostatic forces) rather than chemical bonding, and testing under identical experimental conditions (particularly the same substrate and excitation wavelength) would help determine if similar high-order bands emerge. Alternatively, employing a thiol molecule recognized for its lack of surface photochemical reactions for comparison would be beneficial.

We thank the reviewer for this insightful comment. We agree that the appearance of high-order SERS bands is not expected to be limited exclusively to the 4-ABT/DMAB system and that, in principle, other strongly adsorbed molecules may also exhibit such features under appropriate conditions.

However, based on our results, we would like to emphasize that a key requirement for the observation of high-order transitions is the presence of an electronic transition that overlaps with the excitation wavelength and/or the inelastically scattered photons. This condition can be fulfilled either by molecules that inherently possess such an absorption band (e.g., methylene blue) or by

molecules whose electronic structure is modified upon interaction with the metal surface, leading to the formation of a molecule–surface complex with a shifted absorption band (as in the case of 4-ABT, benzenethiol, or acetaminophen).

To support this interpretation, we have performed additional measurements on methylene blue adsorbed on Ag substrates. In this case, high-order transitions are clearly observed for excitation wavelengths of 532 and 633 nm, which overlap with the molecular absorption band (centered around ~620 nm), while they are significantly weaker for 785 nm excitation. This behavior is consistent with the proposed mechanism and demonstrates that high-order transitions can also arise in systems where adsorption is not necessarily dominated by covalent bonding.

Furthermore, in response to a similar comment from Reviewer 1, we have included measurements on benzenethiol as a non-reactive thiol reference system. These results confirm that high-order transitions can be observed even in the absence of photoinduced chemical transformation, provided that the necessary resonance conditions are met.

We have clarified this point in the revised manuscript to better distinguish between the roles of adsorption, electronic structure, and resonance conditions in the formation of high-order transitions:

"*Interestingly, high-order transitions in SERS have previously been reported mainly for resonant structures. This behavior can be further illustrated using methylene blue (MB), which typically interacts with plasmonic surfaces via non-covalent interactions involving its aromatic system and exhibits a strong electronic transition around 620 nm (Figure S13).*[27] *This is consistent with spectra measured on our silver substrate, where high-order transitions are most pronounced for excitation wavelengths of 532 nm and 633 nm (Figures S14–S15), i.e., under conditions where the excitation significantly overlaps with the molecular absorption band. In contrast, for 785 nm excitation, which overlaps only weakly or not at all with this transition, the high-order features are considerably less intense (Figure S16). In our case, however, the calculated absorption spectrum of 4-ABT shows its most intense band at 229 nm, which is too far from any of the applied excitation wavelengths. Nevertheless, 4-ABT contains a thiol group with a strong affinity to plasmonic surfaces, favoring the formation of covalently bound surface complexes. This likely*

induces a red shift of the HOMO–LUMO transition into the visible range, as demonstrated in our previous study and shown in Figure S17.[12] *Importantly, such a shift is likely to occur not only for 4-ABT, but also for its potential photochemical product, 4,4'-DMAB (Figure S18), as well as for BT (Figure S19), as they all possess a thiol group. This indicates that the maxima of the absorption bands of these surface complexes are shifted to the region between 500 nm and 600 nm, resulting in overlap with the excitation wavelengths used and thereby explaining the presence of high-order transitions.*"

2. Why does the relative intensity ratio of the high-order bands (Figure 3e) vary with wavelength? Is this variation directly attributable a resonance Raman effect of the DMAB molecule, or dose it relates to charge-transfer resonance with the metal?

We thank the reviewer for this insightful questions. We agree that both molecular resonance Raman effects and charge-transfer interactions with the metal can influence the relative intensities of Raman bands and may contribute to variations observed in the high-order region. However, based on our results, we believe that the dominant factor governing the changes in relative intensities of the high-order bands is the ongoing photoinduced transformation of 4-ABT into DMAB. As different excitation wavelengths lead to different extents of this transformation, the relative intensities of DMAB-related fundamental bands vary accordingly, which is subsequently reflected in the high-order transition region.

This interpretation is supported by measurements performed on benzenethiol, which does not undergo comparable photochemical transformation under the studied conditions. In this case, the relative intensities of both fundamental and high-order bands remain essentially unchanged across different excitation wavelengths, indicating that resonance-related effects alone do not lead to significant variations of the type observed for the 4-ABT/4,4´-DMAB system. Therefore, while resonance Raman and charge-transfer effects likely contribute to minor variations in band intensities, the pronounced changes observed in Figure 3 are primarily attributed to changes in the chemical composition of the system.

We have clarified this point in the revised manuscript:

"*To further confirm that the changes in the relative intensities of high-order transition bands are associated with the ongoing photochemical reaction, additional measurements were performed*

using the same Ag-enhancing substrate and benzenethiol (BT), a molecule not expected to undergo photochemical transformation under the applied conditions. The results, obtained using excitation wavelengths of 532 nm, 633 nm, and 785 nm (Figures S7–S9), show that the relative intensities of individual fundamental bands do not significantly vary with excitation wavelength, supporting the assumption that no photochemical transformation occurs in this case."

"*In addition, minor variations in band area ratios may also arise from the spectral positions of the inelastically scattered photons relative to the maxima of the surface plasmon resonance.*"

3. The authors mention that the surface complex may induce a red-shift in the HOMO-LUMO gap but fail to provide experimental (e.g., UV-Vis) or computational evidence to support the existence of an electronic resonance for this complex at the employed excitation wavelengths.

We thank the reviewer for this important comment. We agree that supporting evidence for the proposed shift in the electronic structure of the molecule–surface complex should be clearly provided. In our previous work (Ref. 12), we have already reported computational results demonstrating that the formation of the molecule–surface complex leads to a red-shift of the absorption band into the visible region. However, we acknowledge that this point was not sufficiently emphasized in the present manuscript.

To address this, we have now included additional supporting data in the revised version. Specifically, we have added calculated UV–Vis spectra of the relevant molecule–surface complexes (Figures S17–19 and Figure S2), which illustrate the shift of the absorption band toward the spectral region corresponding to the employed excitation wavelengths. These results support the presence of an electronic resonance under the experimental conditions and are consistent with the proposed mechanism for the enhancement of higher-order transitions.

The manuscript has been revised accordingly to make this point clearer:

"*In our case, however, the calculated absorption spectrum of 4-ABT shows its most intense band at 229 nm, which is too far from any of the applied excitation wavelengths. Nevertheless, 4-ABT contains a thiol group with a strong affinity to plasmonic surfaces, favoring the formation of covalently bound surface complexes. This likely induces a red shift of the HOMO–LUMO transition into the visible range, as demonstrated in our previous study and shown in Figure S17.*[12] *Importantly, such a shift is likely to occur not only for 4-ABT, but also for its potential*

photochemical product, 4,4'-DMAB (Figure S18), as well as for BT (Figure S19), as they all possess a thiol group. This indicates that the maxima of the absorption bands of these surface complexes are shifted to the region between 500 nm and 600 nm, resulting in overlap with the excitation wavelengths used and thereby explaining the presence of high-order transitions."

4. The authors must explore the underlying physical mechanism responsible for the enhancement of high-order bands more thoroughly. Is it merely a byproduct of pure electromagnetic field enhancement, or is it specifically linked to chemical enhancement (charge transfer)?

We thank the reviewer for this important question. We agree that a clearer discussion of the underlying enhancement mechanism is beneficial. Based on our results, the observation of high-order transitions cannot be attributed solely to electromagnetic enhancement. While the electromagnetic mechanism is essential for providing sufficient overall signal intensity, it does not by itself explain the selective appearance and variability of high-order bands.

Our findings indicate that chemical enhancement, particularly involving resonance effects, plays a key role. Specifically, the presence of an electronic transition that overlaps with the excitation wavelength and/or the inelastically scattered photons appears to be a critical factor. This condition can be fulfilled either by the intrinsic absorption of the molecule (as in the case of methylene blue) or by the formation of a molecule–surface complex, which modifies the electronic structure and shifts the absorption band into the relevant spectral region (as observed for 4-ABT/DMAB and benzenethiol). This interpretation is supported by the comparative experiments presented in this work, including the absence of high-order transitions on Au substrates, the wavelength-dependent behavior, and the results obtained for non-reactive reference systems.

We have revised the manuscript to provide a more explicit and consolidated discussion of this mechanism:

"*Importantly, all these observations further suggest that, while electromagnetic enhancement is necessary to achieve sufficient signal intensity, it is not sufficient to explain the appearance of high-order transitions. Instead, chemical enhancement mechanisms, particularly those involving resonance with electronic transitions of the molecule or molecule–surface complex, appear to play a decisive role. Under such conditions, the presence of resonance governs both the visibility and the relative intensity of high-order bands. The ongoing chemical transformations are manifested*

through the dependence of the high-order bands' relative intensities on the excitation wavelength used, reflecting different reaction kinetics under different excitation wavelengths. This is further confirmed by additional measurements performed on acetaminophen (AAP), which is also capable of forming photoactive covalently bound surface complexes (Supplementary Note 1). Furthermore, the fact that the 4-ABT/4,4'-DMAB spectra were measured using a different Raman microscope than BT, MB, and AAP (see Methods section) rules out the possibility that the observed finding are the result of experimental error."

"*By employing different plasmonic metals and model probes, we demonstrate that, although electromagnetic enhancement is required to achieve sufficient signal intensity, the observed high-order bands and their appearance are primarily governed by chemical enhancement mechanisms, particularly those involving resonance with electronic transitions of the molecule or molecule–surface complex, whether present in the native state or induced by covalent interaction with the plasmonic surface.*"

5. It is recommended that the authors apply their current methodology to a novel, controversial molecule-metal system to demonstrate that this approach can effectively address interfacial chemistry problems that are difficult to elucidate using existing techniques, such as conventional SERS or DFT. Otherwise, the manuscript may be better suited for submission to a specialized journal focused on spectroscopic characterization.

We thank the reviewer for this suggestion. We agree that applying the proposed methodology to additional, more complex or controversial molecule–metal systems would be an interesting direction for future research.

However, the primary aim of the present work is to establish and demonstrate a general approach for the interpretation of high-order SERS transitions and to elucidate the key factors governing their appearance. In this context, we believe that the systems studied here provide a suitable and sufficiently representative basis for developing and validating this methodology.

We further note that the results obtained for multiple systems within this work (including reactive and non-reactive molecules, as well as different substrates) already demonstrate the broader applicability of the proposed approach.

A systematic application to more complex interfacial systems, while certainly valuable, would require a substantially expanded study and is therefore beyond the scope of the present manuscript. We believe that the general insights provided here will facilitate such future investigations.

Response to the Reviewers and Editorial Revisions

Responses to the Reviewers

Reviewer #1 (Remarks to the Author):

The authors carried out the suggested additional measurements and responded thoroughly to my comments. I am somewhat surprised that DMAB modes were not observed in the gold samples, but their conclusions remain consistent.

I therefore recommend the publication of this article.

Reviewer #2 (Remarks to the Author):

The authors have revised the manuscript and adequately addressed all reviewer comments. The quality of the manuscript is now satisfactory. Thus, I recommend the publication of this manuscript in Communications Chemistry.

We sincerely thank both reviewers for their careful evaluation of our manuscript and for their positive comments. We greatly appreciate their recommendation for publication. As no additional scientific revisions were requested, no changes to the manuscript were made in response to the reviewers' reports.

Answers for the editorial revisions

We thank the Editorial Office for the careful assessment of our manuscript and for the helpful suggestions regarding formatting and presentation. The requested editorial revisions have been implemented. Detailed responses to each editorial comment are provided in the Editorial Revision checklist submitted together with the revised manuscript.
