# Supplementary material for: Reconstructing high-order SERS transitions in an overlooked spectral region reveals surface complex formation
Source: Commun Chem. 2026 Jul 17;9:353. doi: 10.1038/s42004-026-02136-x (PMC13748603; doi:10.1038/s42004-026-02136-x)
Supplement: Supplementary file 2 [file 42004_2026_2136_MOESM2_ESM.pdf]

Supporting Information

Reconstructing High-Order SERS Transitions in an Overlooked Spectral Region Reveals Surface Complex Formation

Ivan Kopal[1,2*], Matěj Kmetík[1,3], Pavel Matějka[1], Marek Piliarik[2], Marcela Dendisová[1]

[1] Department of Physical Chemistry, University of Chemistry and Technology Prague,

Technická 5, 160 00 Prague 6, Czech Republic

[2] Institute of Photonics and Electronics, Czech Academy of Sciences,

Chaberská 1014/57, 182 00 Prague 8, Czech Republic

[3] Institute of Organic Chemistry and Biochemistry, Czech Academy of Sciences,

Flemingovo náměstí 542/2, 160 00 Prague 6, Czech Republic

* Corresponding author: tel.: +420 220 444 040, email: kopali@vscht.cz

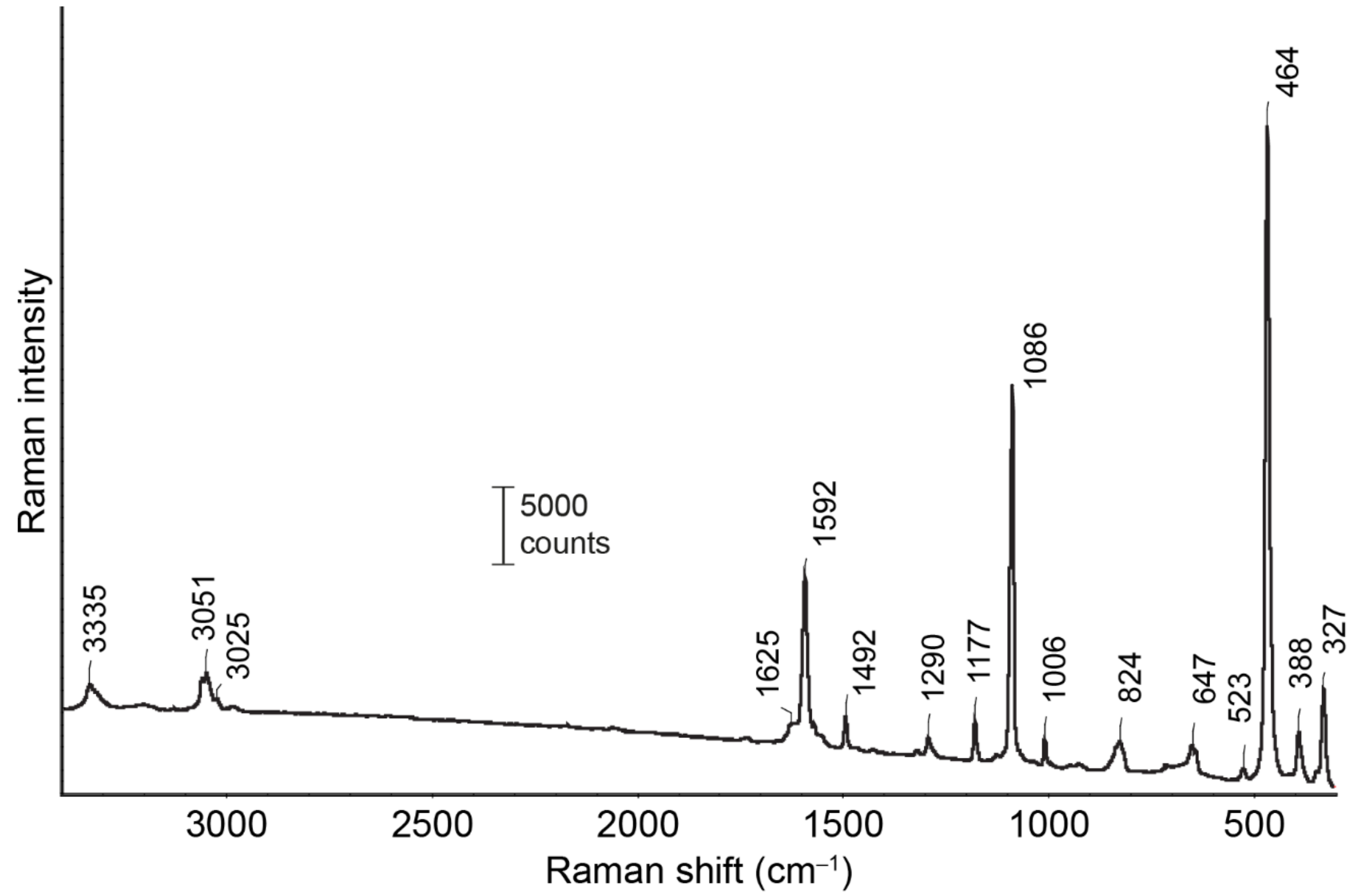

Figure S1 Raman spectrum of crystalline 4-ABT measured using 532 nm excitation (5mW; 10x1s).

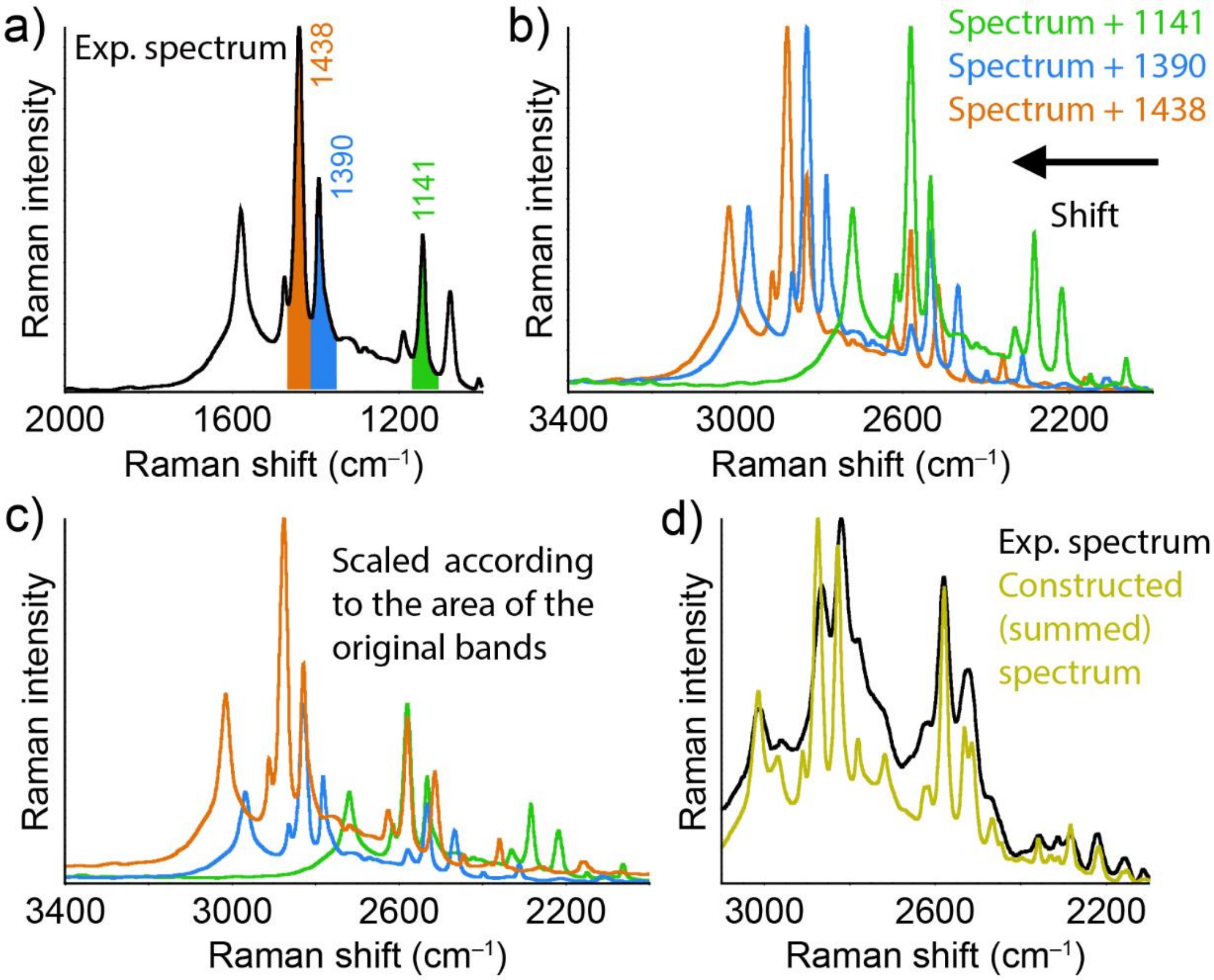

Figure S2 Schematic representation of theoretical high-order spectrum construction from the 455-nm excited spectrum: (a) selected bands; (b) spectrum shifted by each selected Raman shift value (common-scale regime); (c) spectra shifted and scaled according to band areas (common-scale regime); (d) final constructed spectrum obtained by summation of all shifted and scaled spectra compared with the experimentally observed high-order spectrum (full-scale regime).

Table S1 Assignment of fundamental vibrational modes of 4,4´-DMAB.

Raman shift (cm^{-1})		Assignment of the vibrational modes
Experimental	Calculated (Scaling 0.975)	
1577	1585, 1557	ν (C–C)$_{Ar}$, ν (C=C)$_{Ar}$, δ_{ip} (C–H)$_{Ar}$
1473	1467	δ_{ip} (C–H)$_{Ar}$
1438	1419	ν (N=N), ν (C–C)$_{Ar}$, ν (C=C)$_{Ar}$, δ_{ip} (C–H)$_{Ar}$
1390	1380	ν (N=N), ν (C–C)$_{Ar}$, ν (C=C)$_{Ar}$, δ_{ip} (C–H)$_{Ar}$
1329	1331	δ (C–N), ν (C–C)$_{Ar}$, ν (C=C)$_{Ar}$, δ_{ip} (C–H)$_{Ar}$
1280	1292	δ_{ip} (C–H)$_{Ar}$
1188	1188	ν (C–N), δ_{ip} (C–H)$_{Ar}$
1141	1142	ν (C–N), δ_{ip} (C–H)$_{Ar}$
1075	1056	ν (C–S)
1006	1000	δ_{ip} (C–H)$_{Ar}$
947	992	δ_{oop} (C–H)$_{Ar}$
920	898	δ_{ip} (C–N=N–C)
723	746	δ_{oop} (C–N), ν (C–C)$_{Ar}$, ν (C=C)$_{Ar}$, δ_{oop} (C–H)$_{Ar}$
715	730	δ_{oop} (C–N), ν (C–C)$_{Ar}$, ν (C=C)$_{Ar}$, δ_{oop} (C–H)$_{Ar}$
544	559	δ_{oop} (C–N), ν (C–C)$_{Ar}$, ν (C=C)$_{Ar}$, δ_{oop} (C–H)$_{Ar}$

ν – stretching; δ – deformation; ip – in-plane; oop – out-of-plane; Ar – aromatic ring

Table S2 Electrolyte baths and current sequences used for plate platinum targets

	Ag		Au		Cu	
bath	$[Ag(NH_3)_2]^+$		$[Au(NH_3)_4]^{3+}$		$[Cu(NH_3)_4]^{2+}$	
step	*I* [mA]	*t* [min]	*I* [mA]	*t* [min]	*I* [mA]	*t* [min]
1	5	10	5	5	10	10
2	10	5	10	5	20	10
3			15	5	30	10
4					40	10
5					50	10

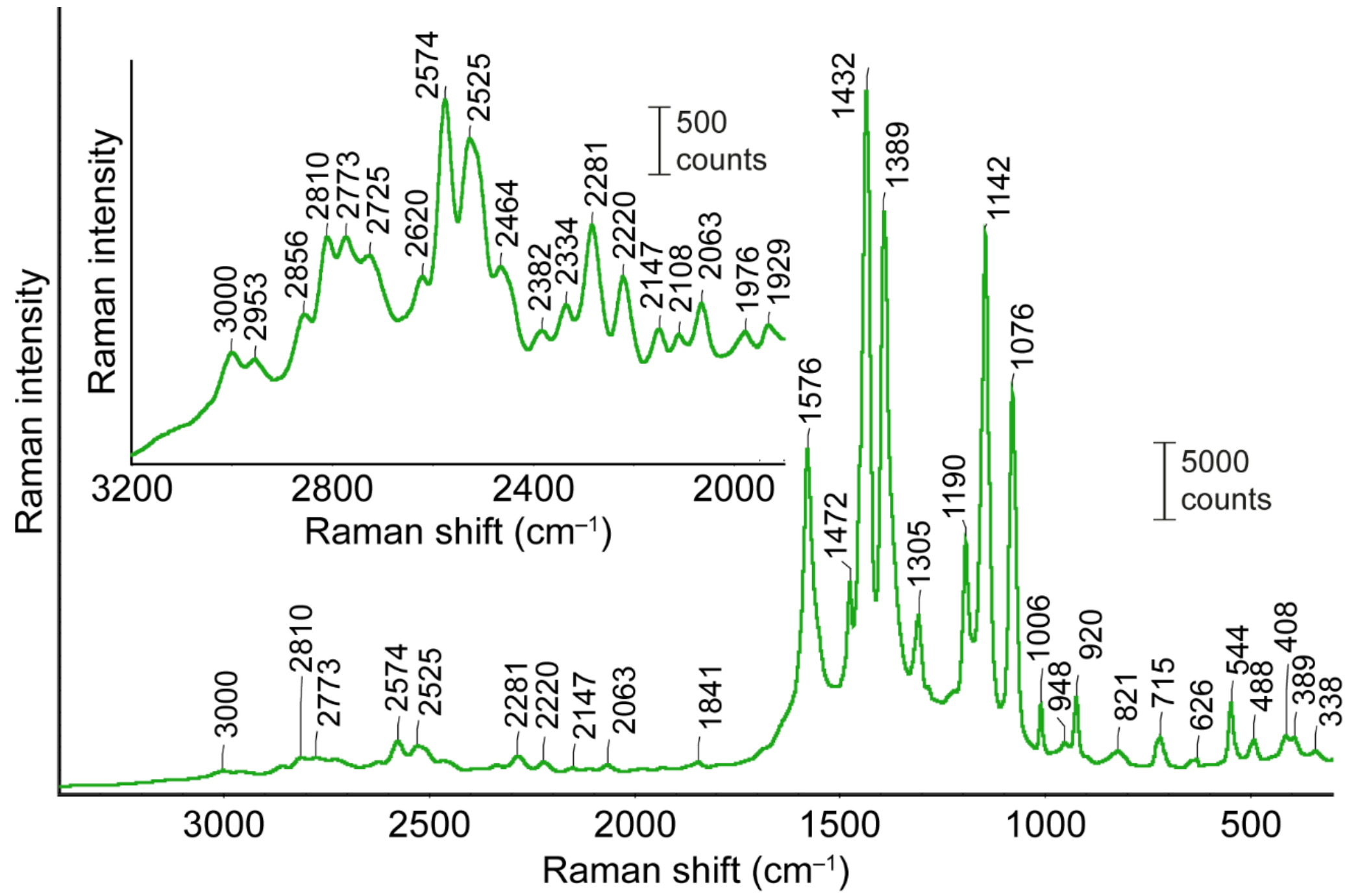

Figure S3 Ag-SERS spectrum of 4-ABT/4,4′-DMAB measured with 532 nm excitation and obtained by averaging 10 individual spectra.

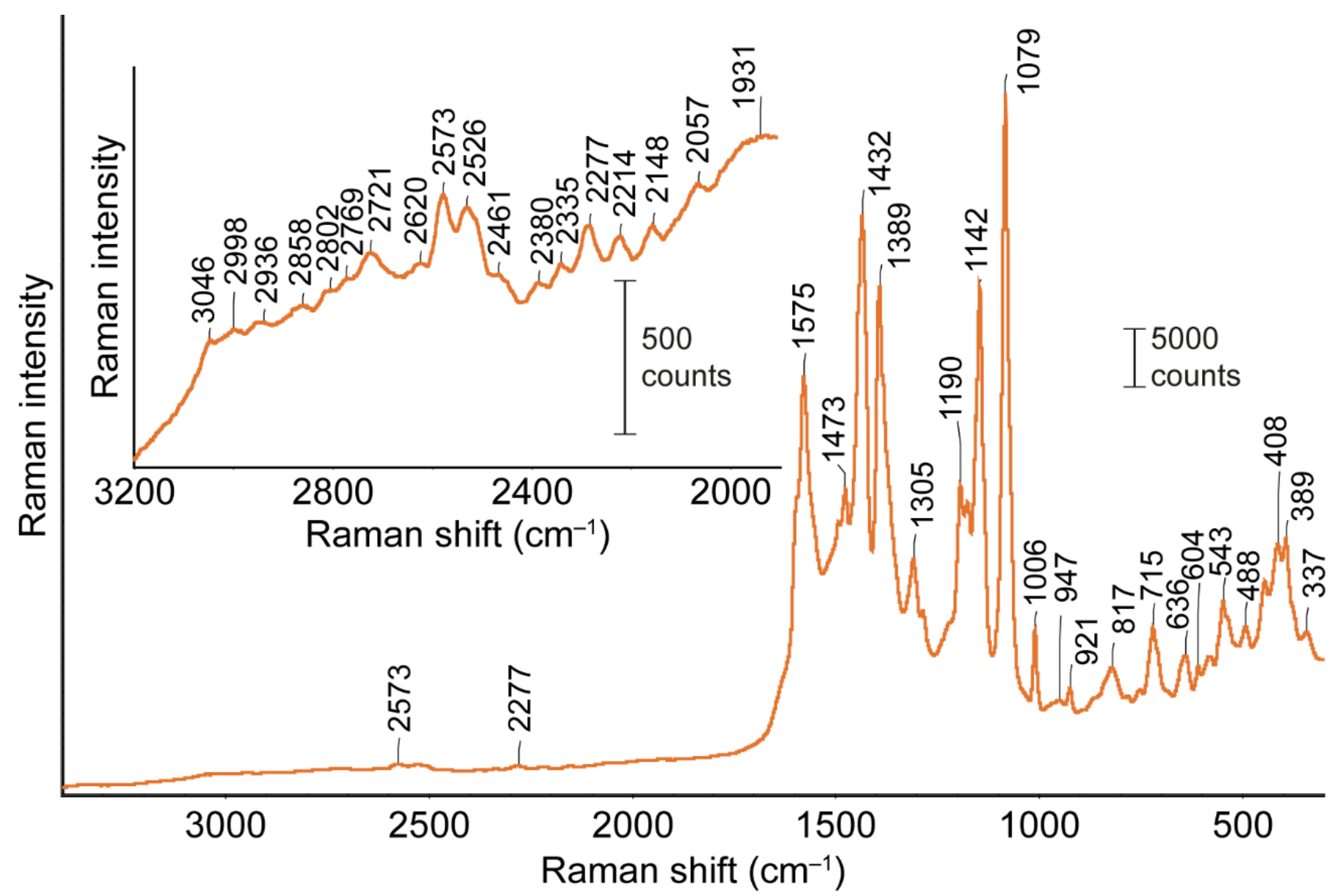

Figure S4 Ag-SERS spectrum of 4-ABT/4,4′-DMAB measured with 633 nm excitation and obtained by averaging 10 individual spectra.

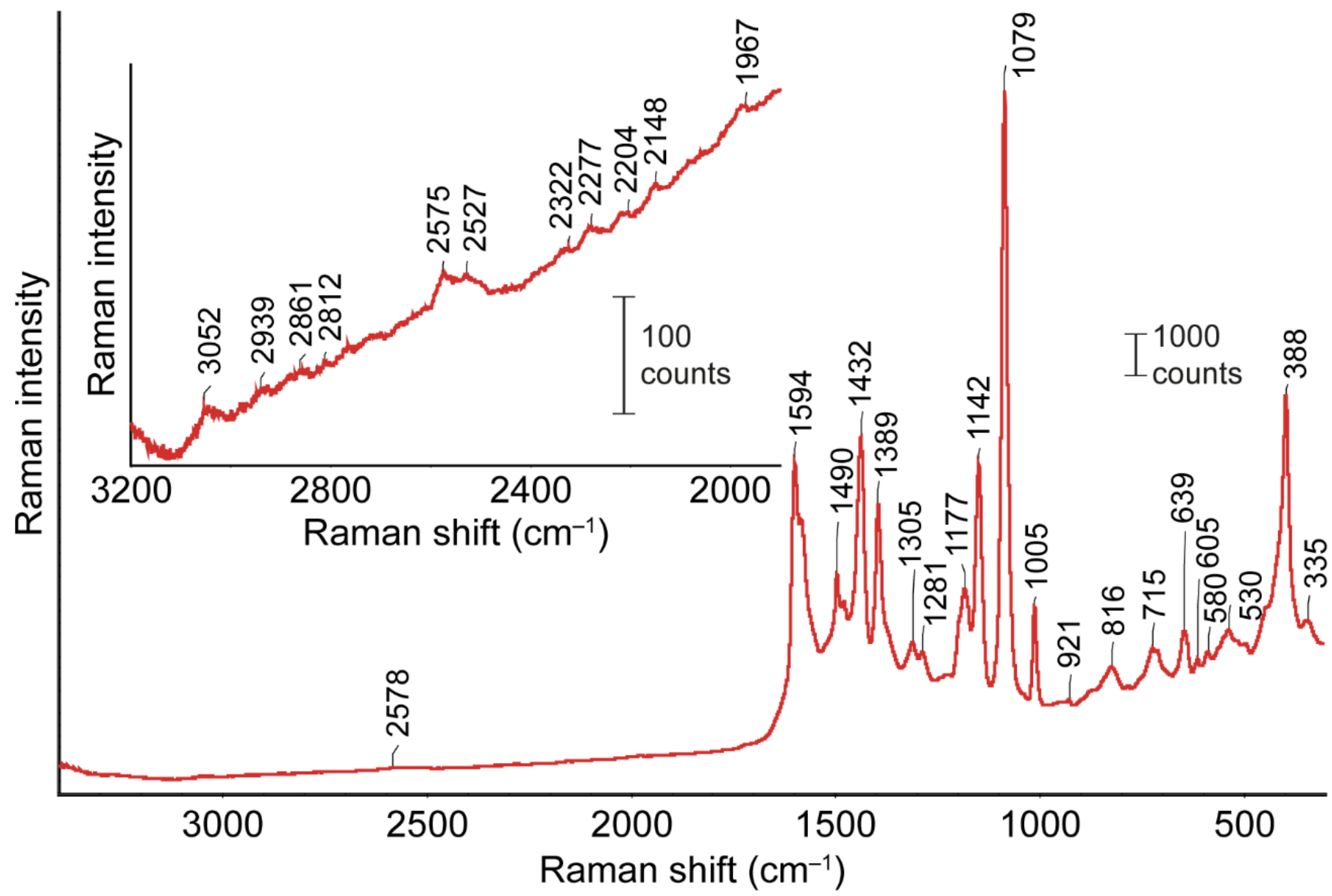

Figure S5 Ag-SERS spectrum of 4-ABT/4,4′-DMAB measured with 780 nm excitation and obtained by averaging 10 individual spectra.

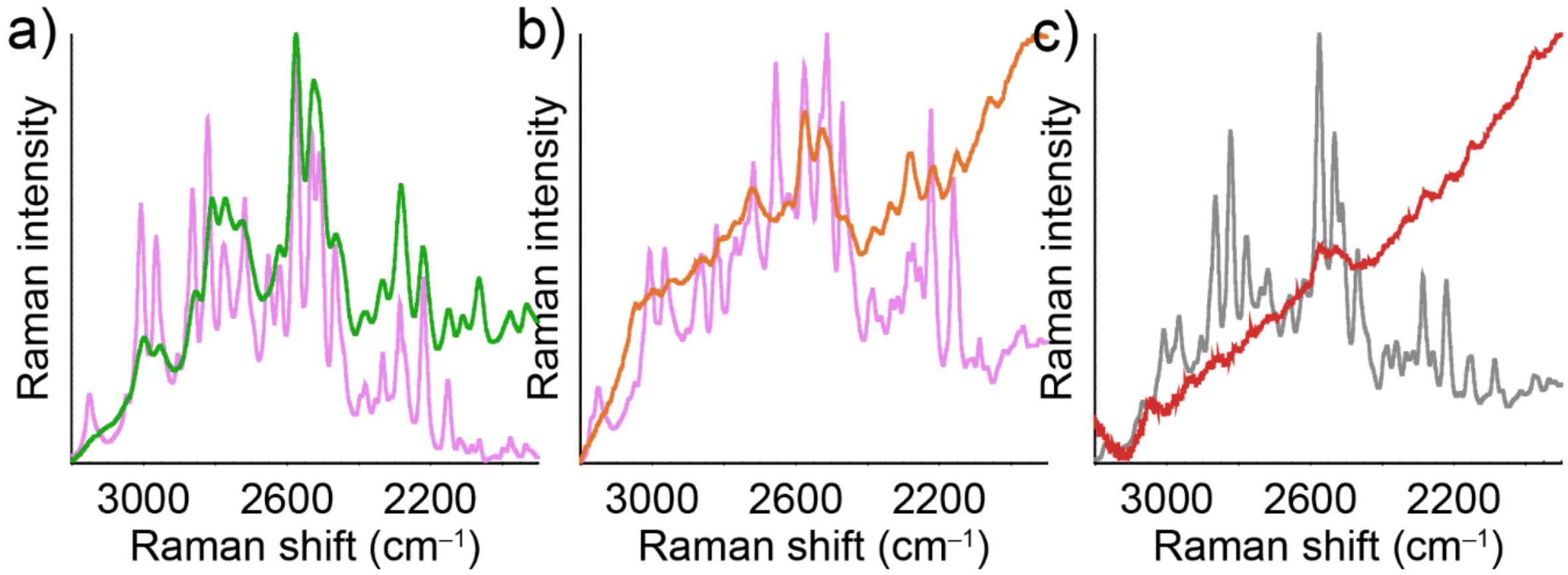

Figure S6 Reconstruction of high-order SERS transitions at different excitation wavelengths. Comparison: a) of spectra constructed from the sum of all spectra shifted to higher Raman shift values according to individual band positions and scaled according to the area of the original bands with the experimental spectrum measured with 532 nm excitation; b) of spectra constructed from the sum of all spectra shifted to higher Raman shift values according to individual band positions and scaled according to the area of the original bands with the experimental spectrum measured with 633 nm excitation; c) of spectra constructed from the sum of all spectra shifted to higher Raman shift values according to individual band positions with the experimental spectrum measured with 780 nm excitation; in the 3200–1900 cm^{-1} region. The spectra are displayed on a full-scale basis.

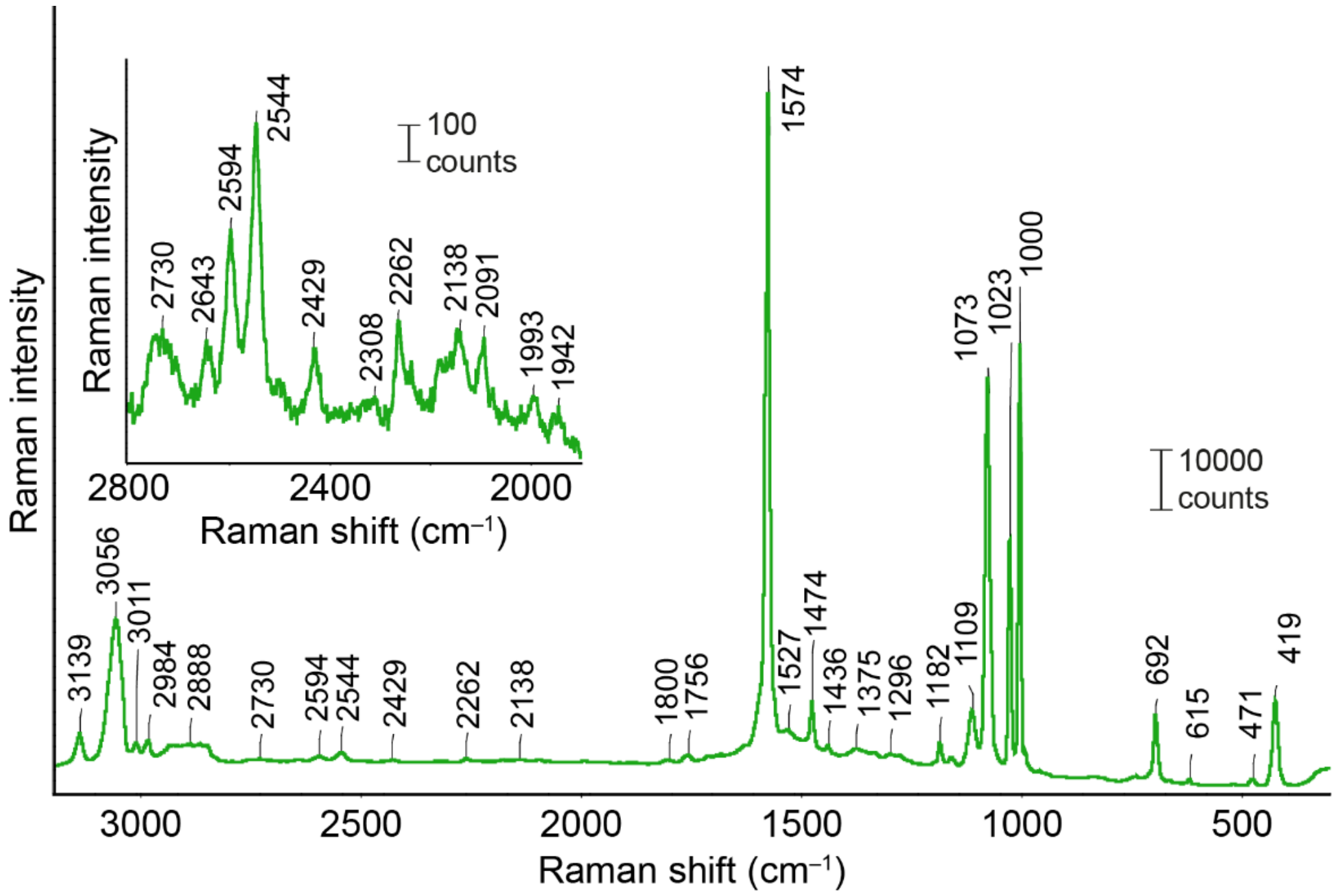

Figure S7 Ag-SERS spectrum of BT measured with 532 nm excitation and obtained by averaging 10 individual spectra.

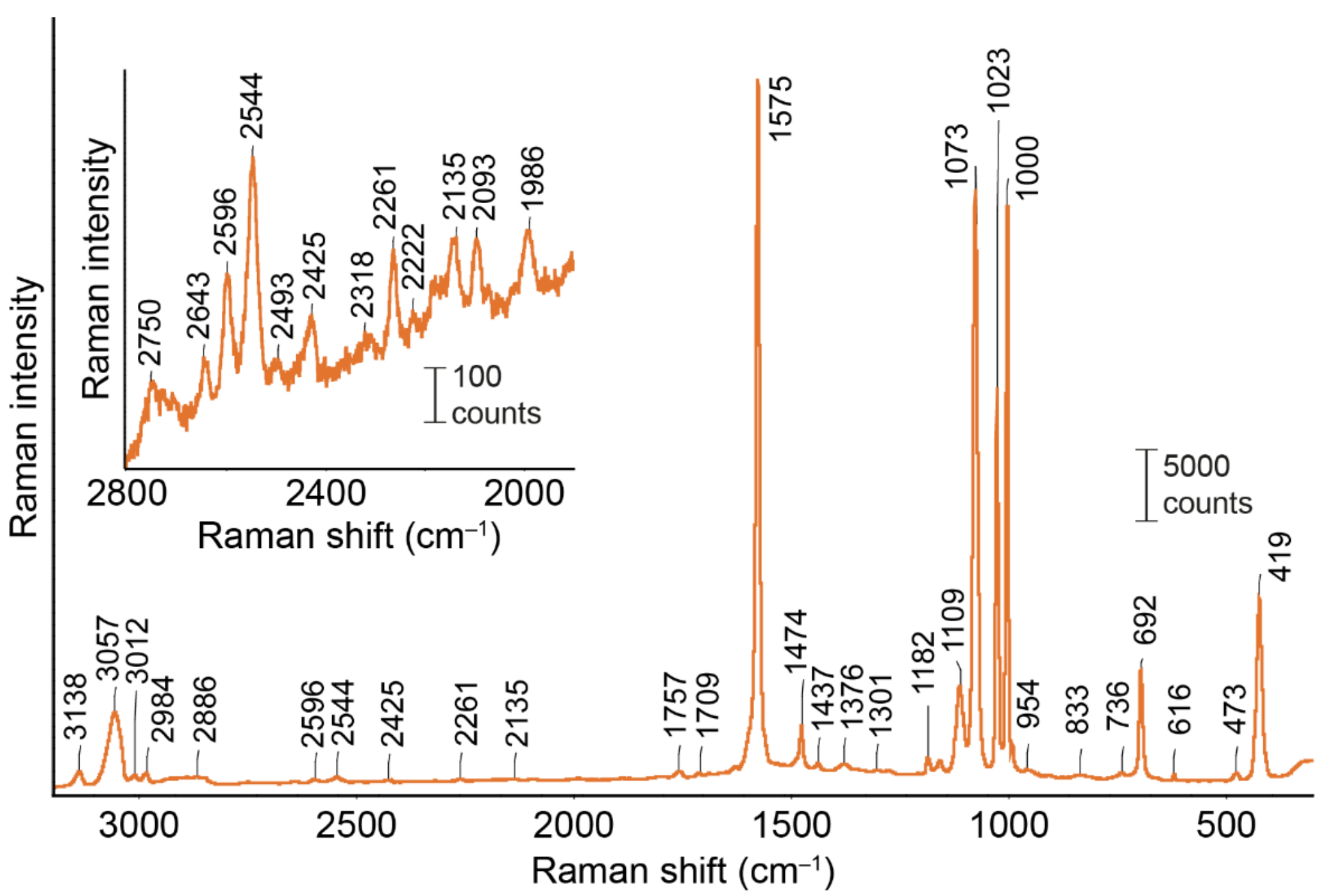

Figure S8 Ag-SERS spectrum of BT measured with 633 nm excitation and obtained by averaging 10 individual spectra.

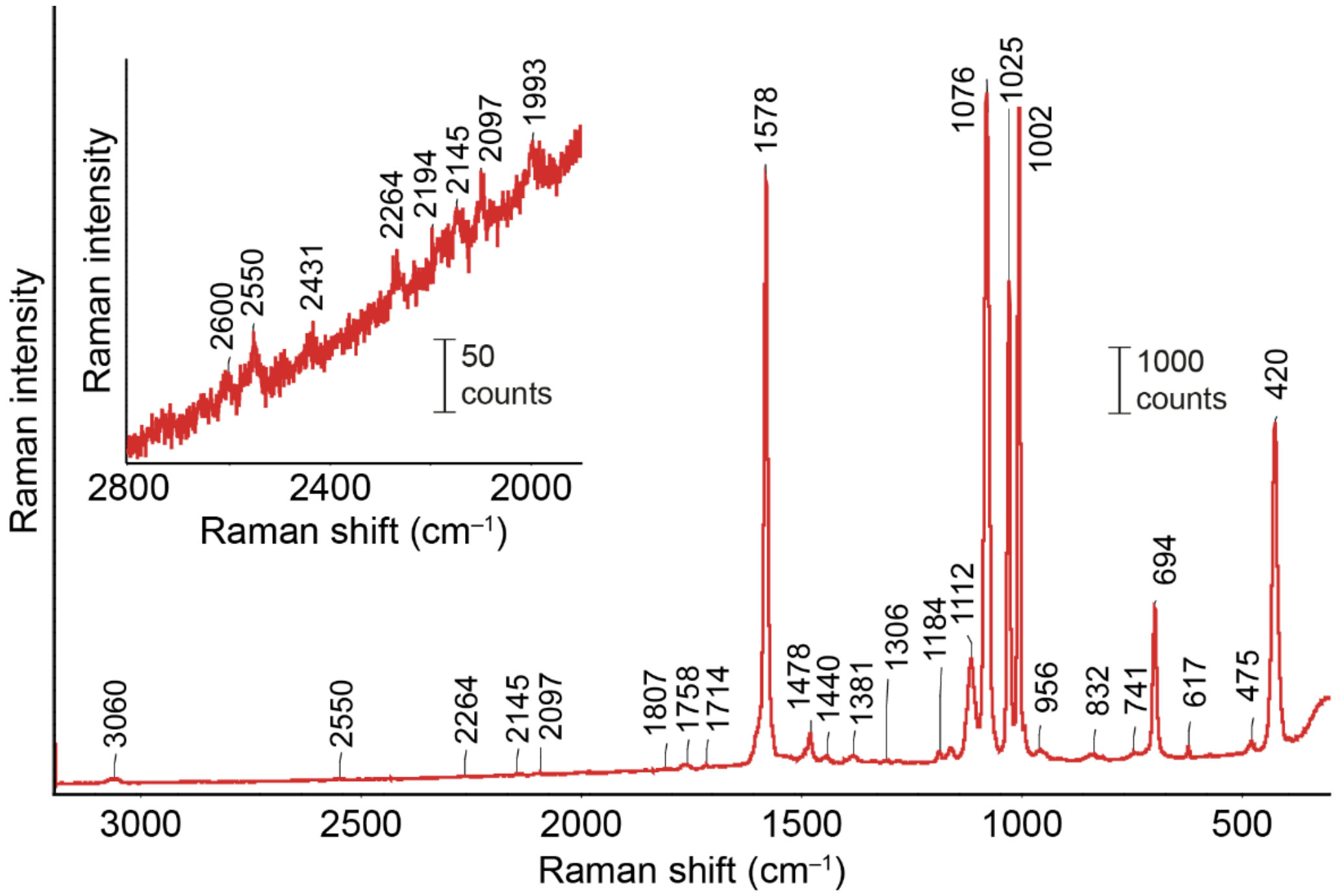

Figure S9 Ag-SERS spectrum of BT measured with 785 nm excitation and obtained by averaging 10 individual spectra.

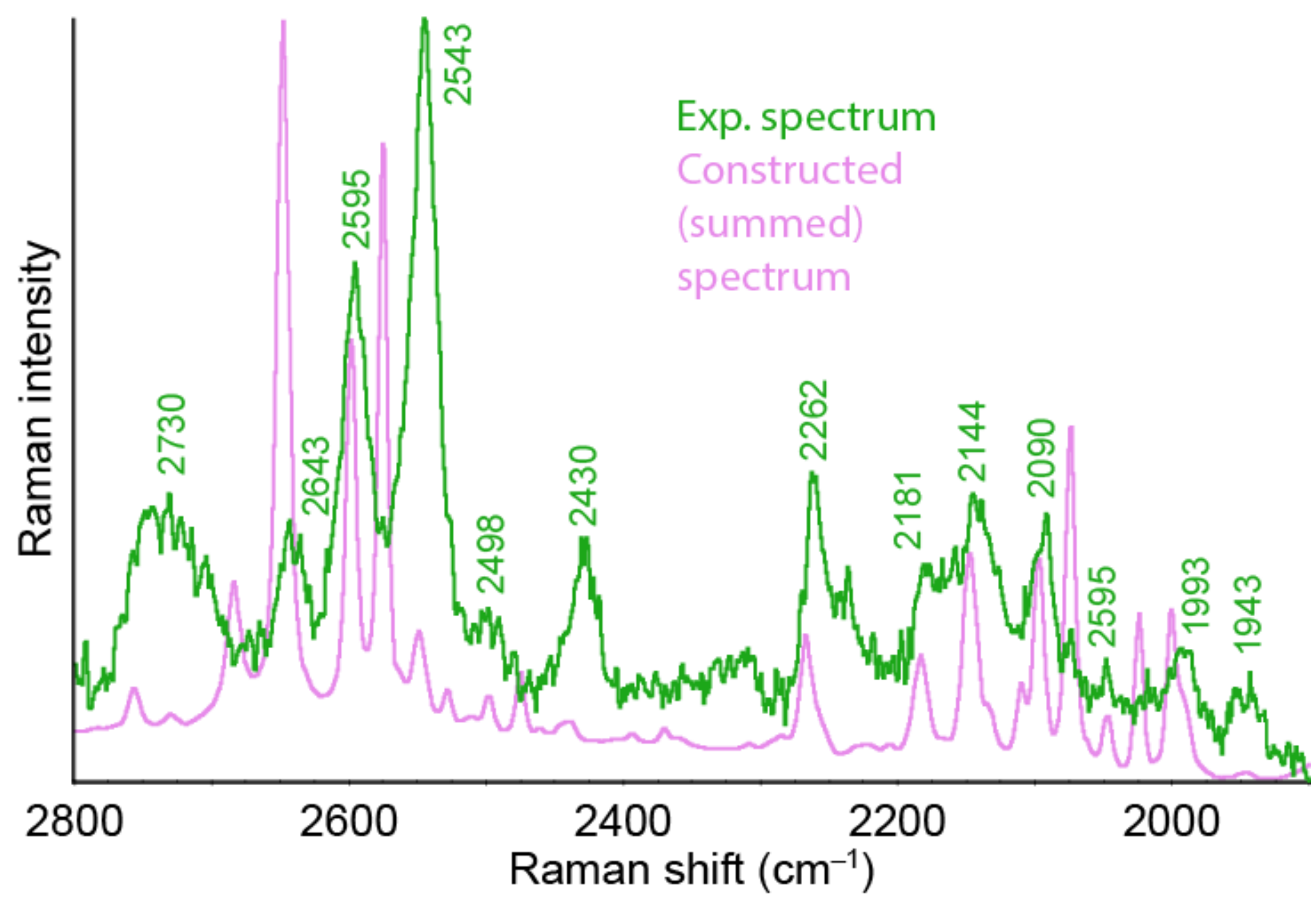

Figure S10 Comparison of the BT spectrum constructed by summing spectra shifted according to the wavenumbers of individual fundamental bands and weighted by their relative areas with the

experimental spectrum measured at 532 nm (3200–1900 cm⁻¹ region). The spectra are displayed on a full-scale basis.

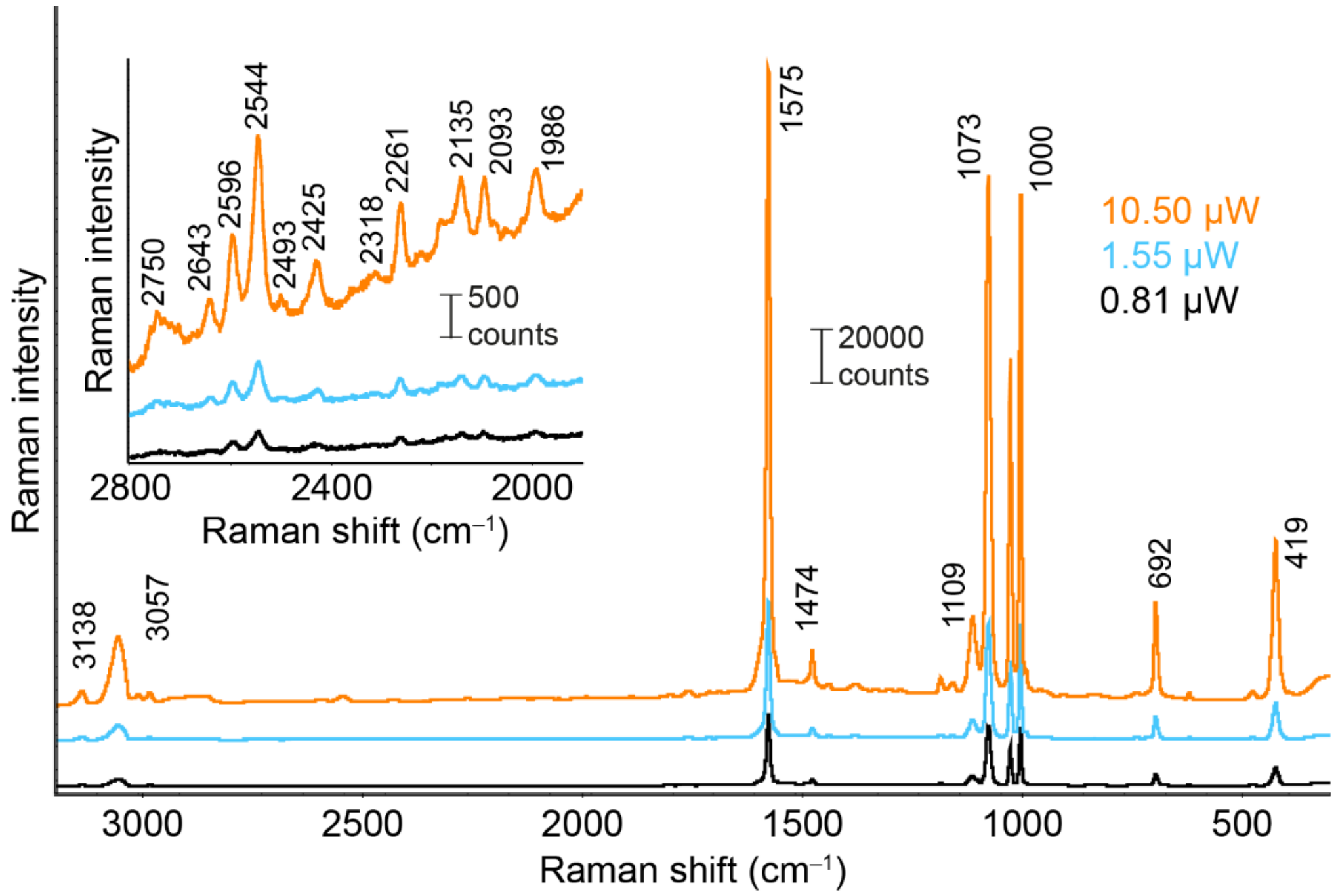

Figure S11 Comparison of Ag-SERS spectra of BT measured at 633 nm excitation using three different laser powers, each obtained by averaging 10 individual spectra. The spectra are displayed on a common intensity scale with an offset.

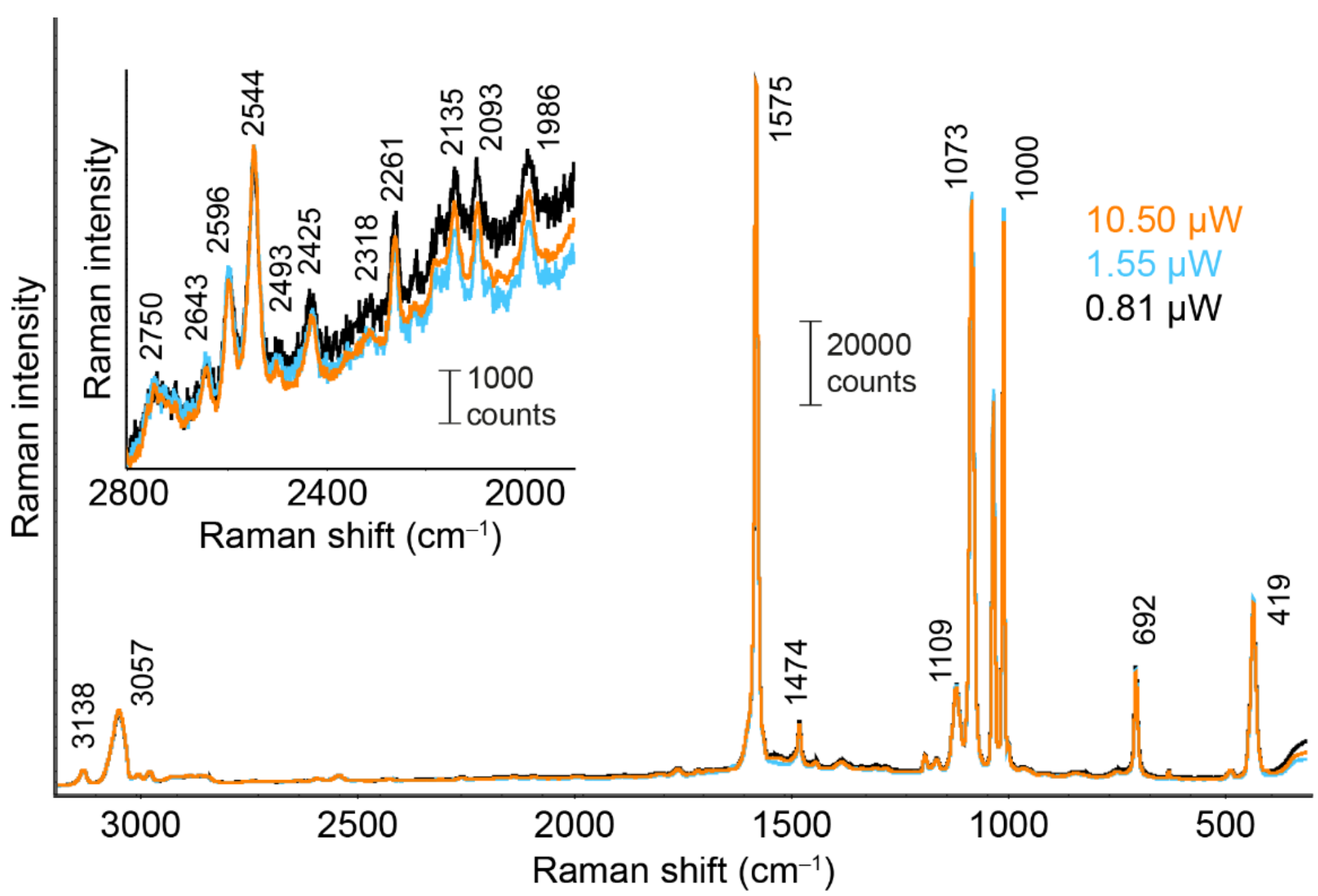

Figure S12 Comparison of Ag-SERS spectra of BT measured at 633 nm excitation using three different laser powers, each obtained by averaging 10 individual spectra. The spectra are displayed on a full-scale basis.

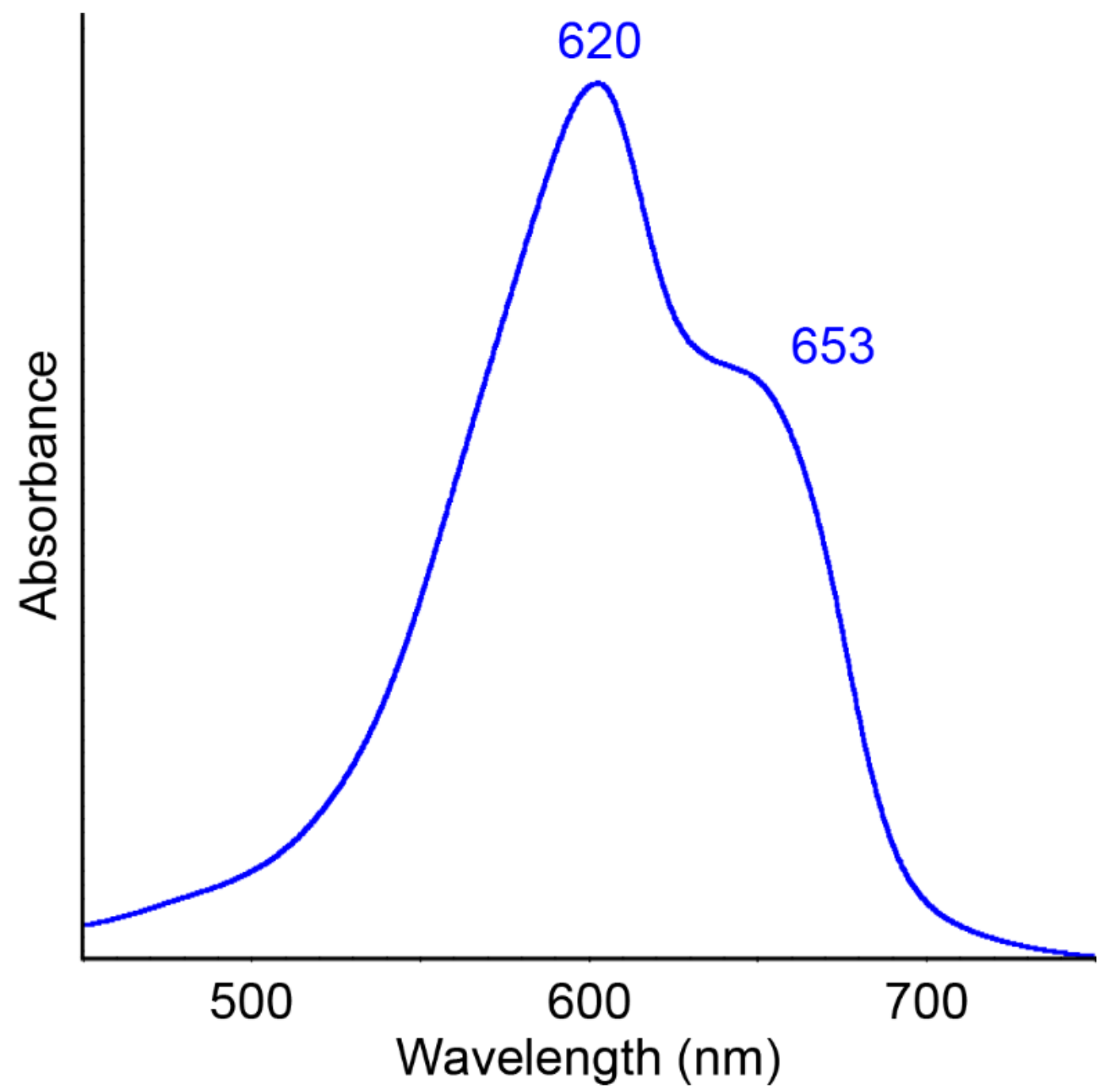

Figure S13 Absorption spectrum of MB (10^{-8} mol/L, aqueous solution).

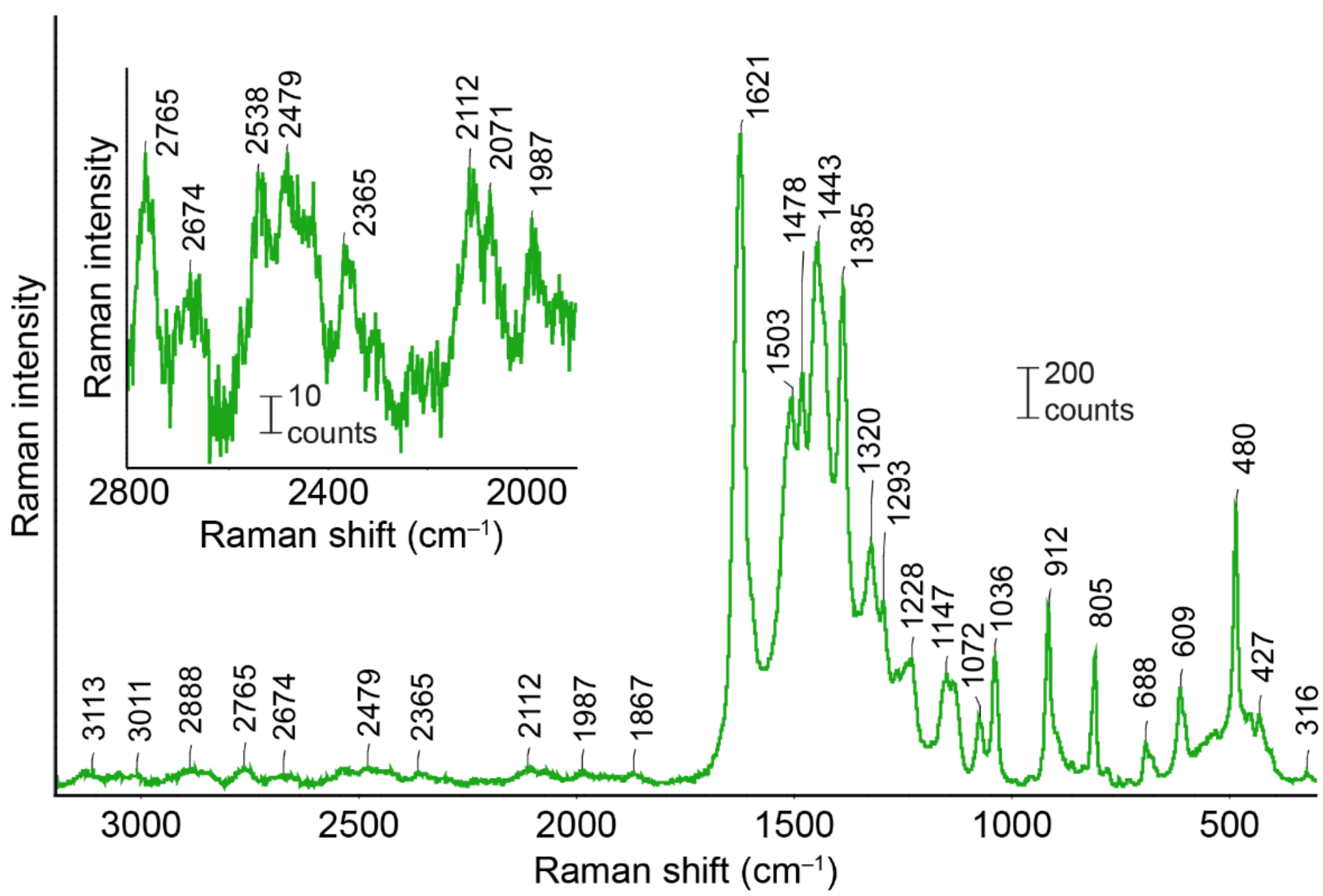

Figure S14 Ag-SERS spectrum of MB measured with 532 nm excitation and obtained by averaging 10 individual spectra.

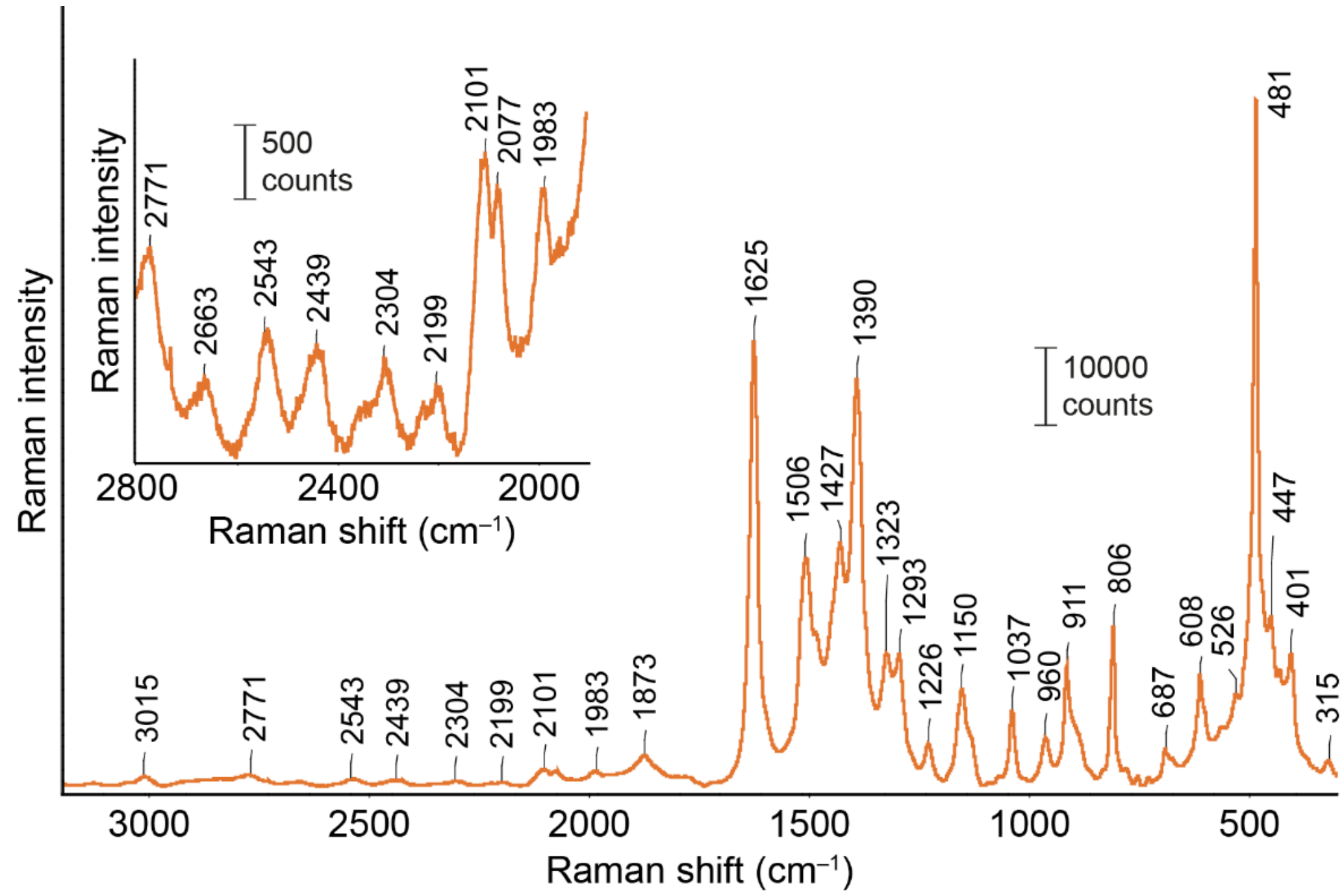

Figure S15 Ag-SERS spectrum of MB measured with 633 nm excitation and obtained by averaging 10 individual spectra.

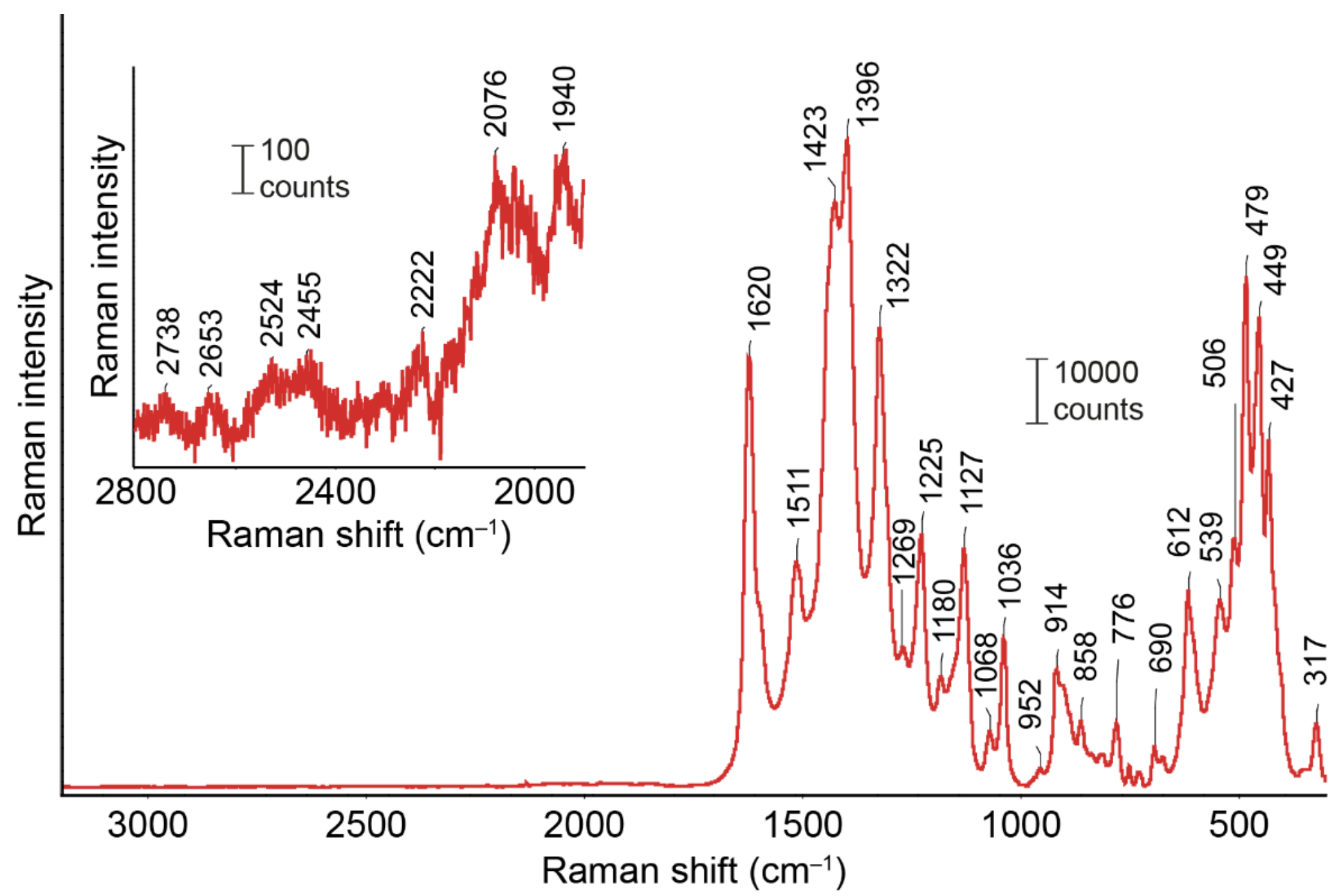

Figure S16 Ag-SERS spectrum of MB measured with 785 nm excitation and obtained by averaging 10 individual spectra.

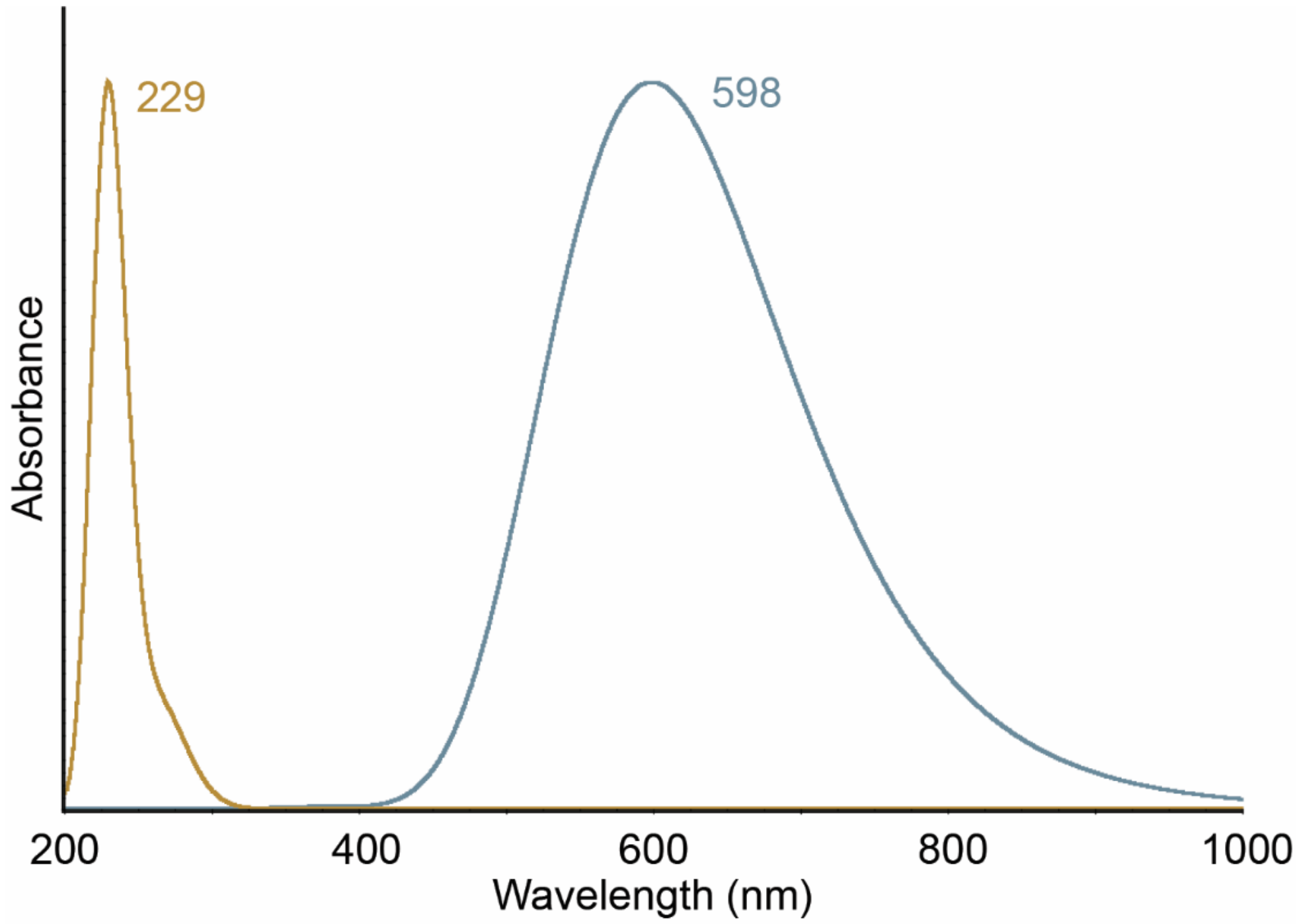

Figure S17 Calculated absorption spectra of 4-ABT in its native state and covalently bound to a silver atom via the thiol group.

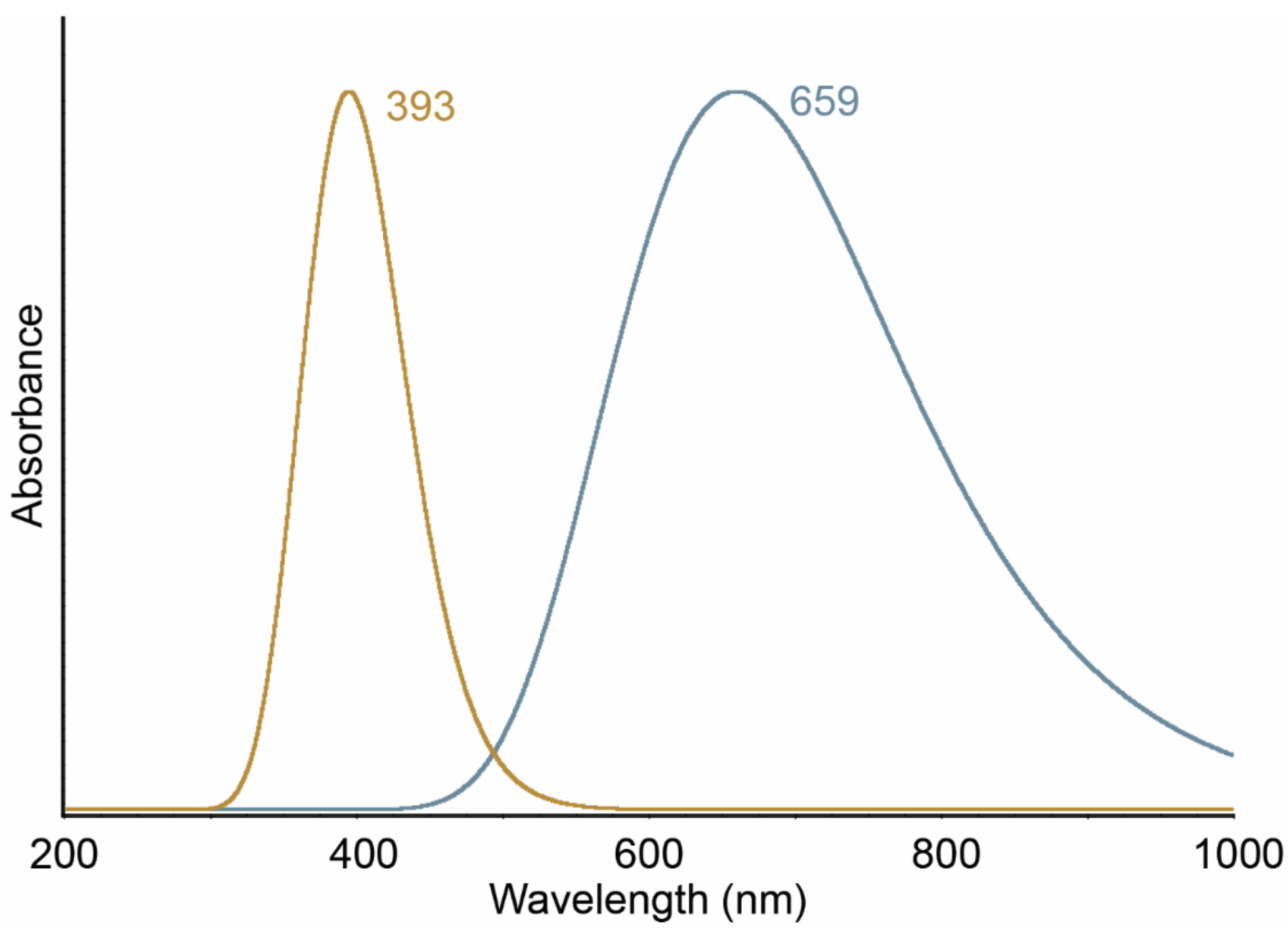

Figure S18 Calculated absorption spectra of 4,4´-DMAB in its native state and covalently bound to a silver atoms via the thiol groups.

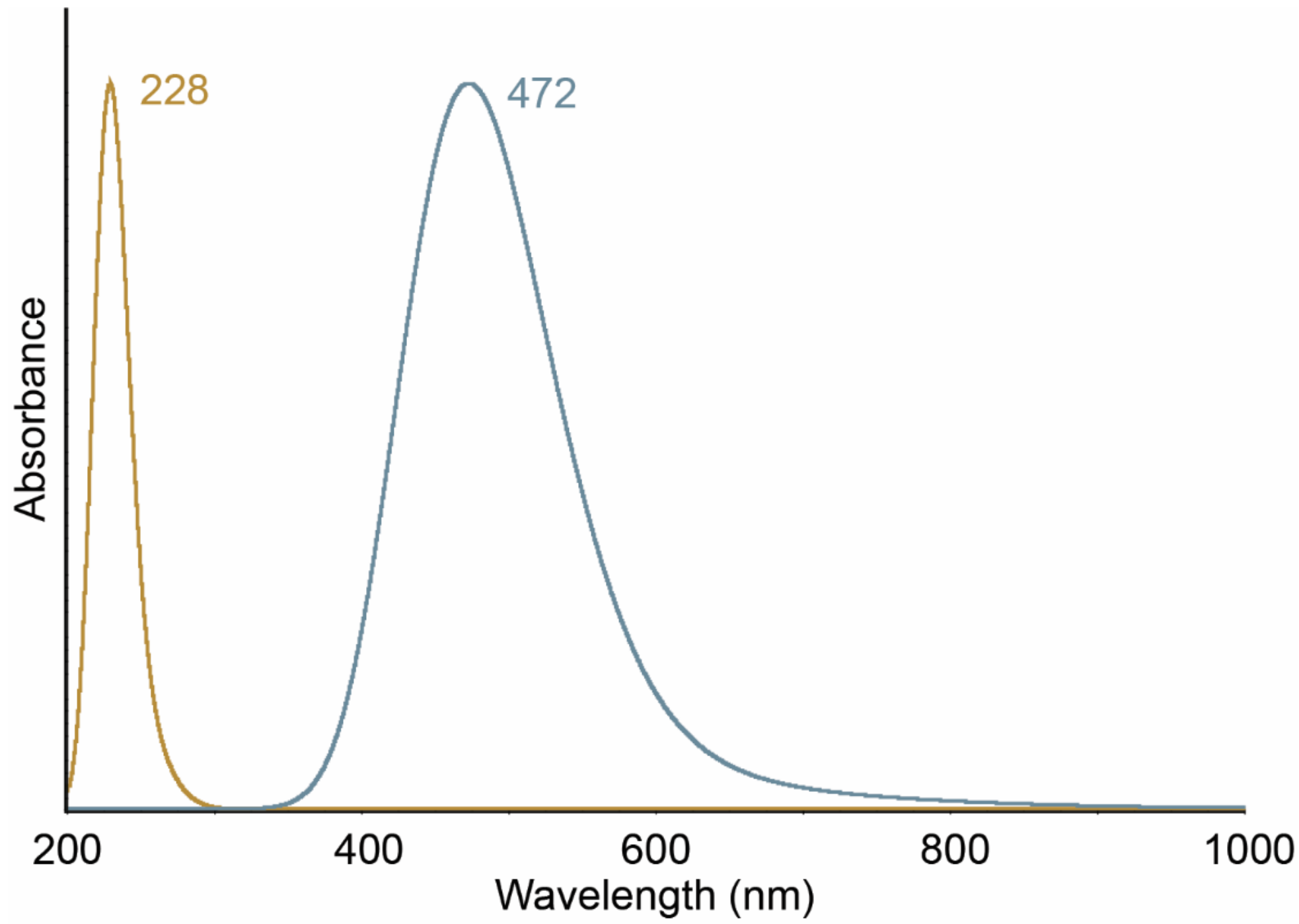

Figure S19 Calculated absorption spectra of BT in its native state and covalently bound to a silver atom via the thiol group.

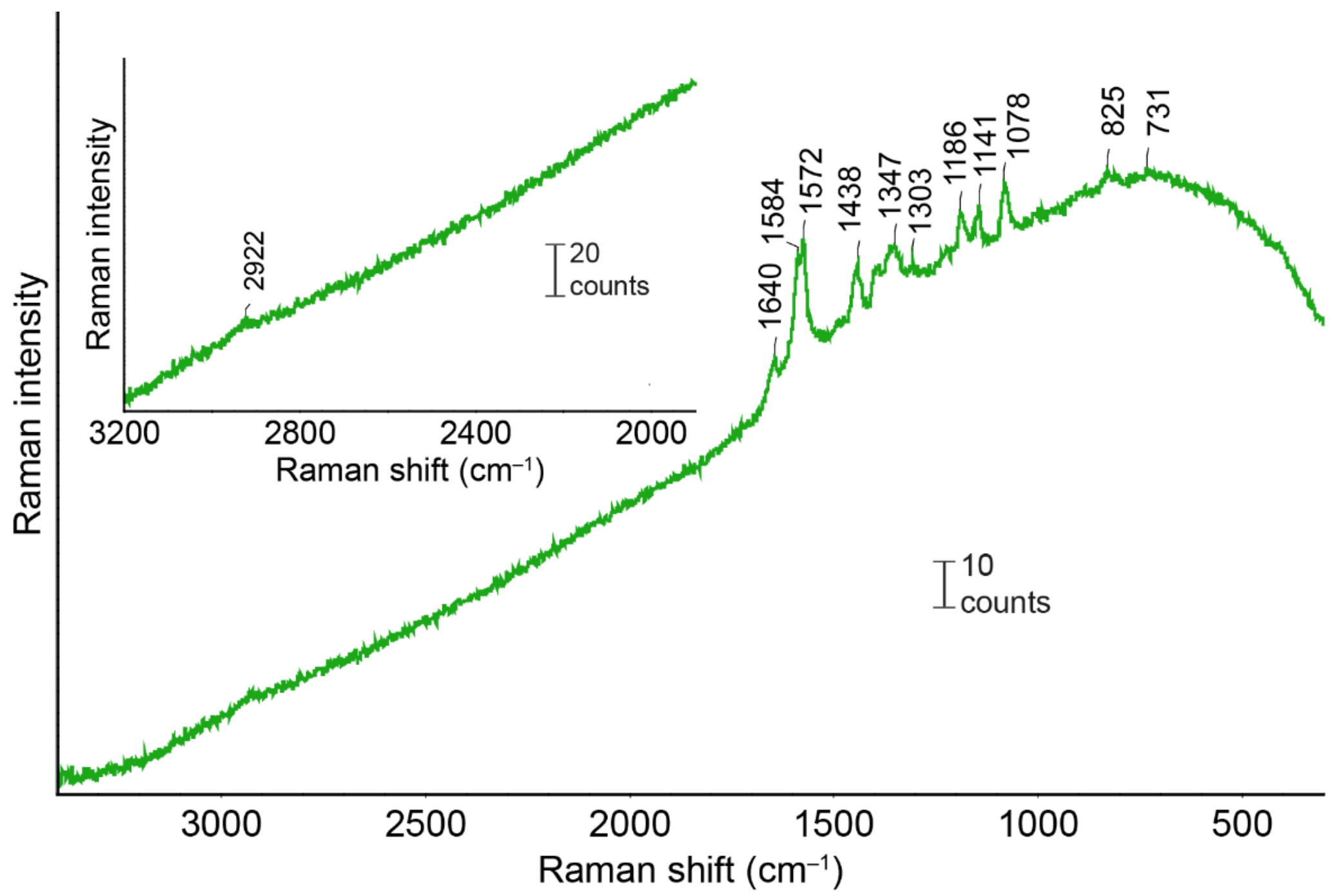

Figure S20 Au-SERS spectrum of 4-ABT/4,4′-DMAB measured with 532 nm excitation and obtained by averaging 10 individual spectra.

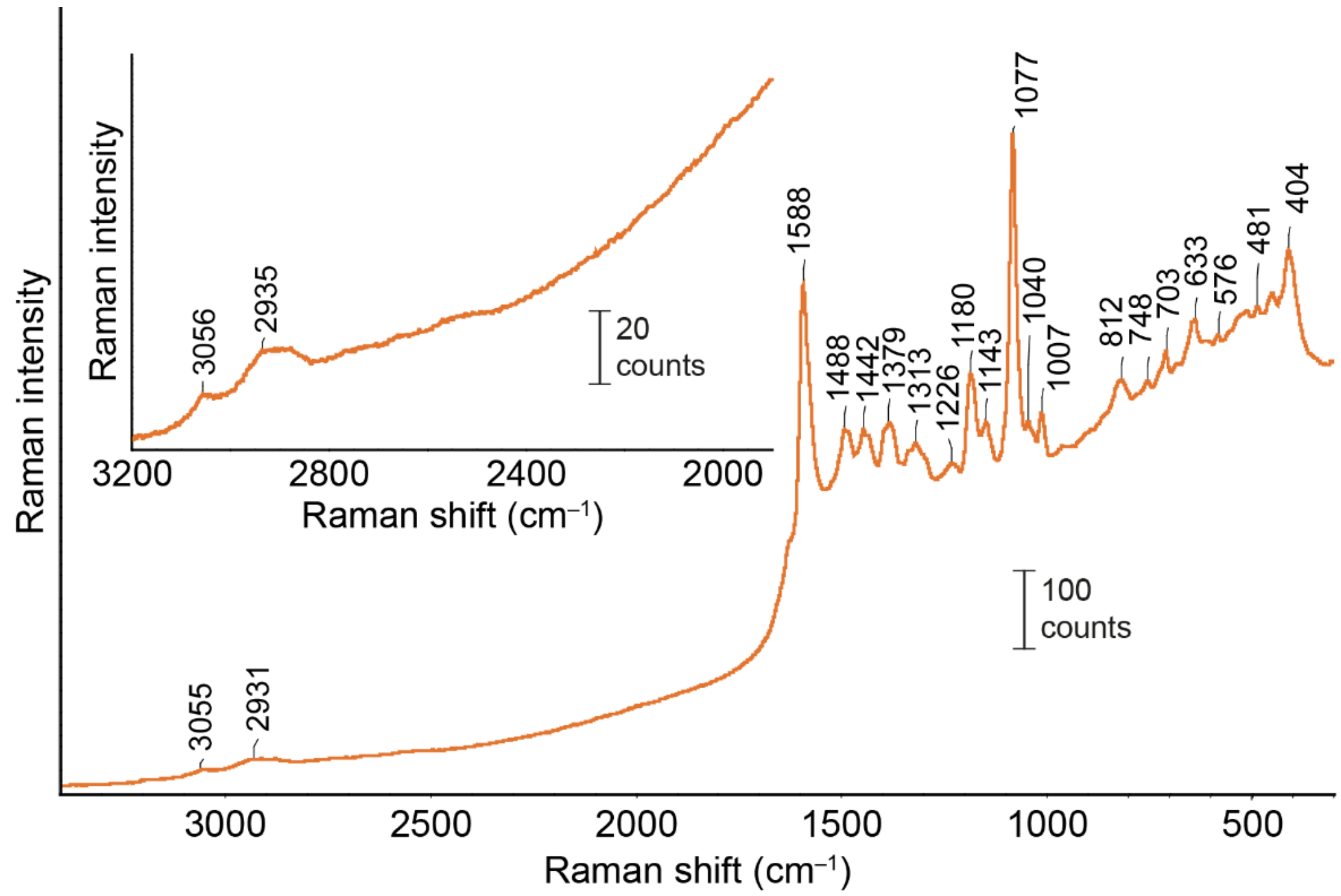

Figure S21 Au-SERS spectrum of 4-ABT/4,4′-DMAB measured with 633 nm excitation and obtained by averaging 10 individual spectra.

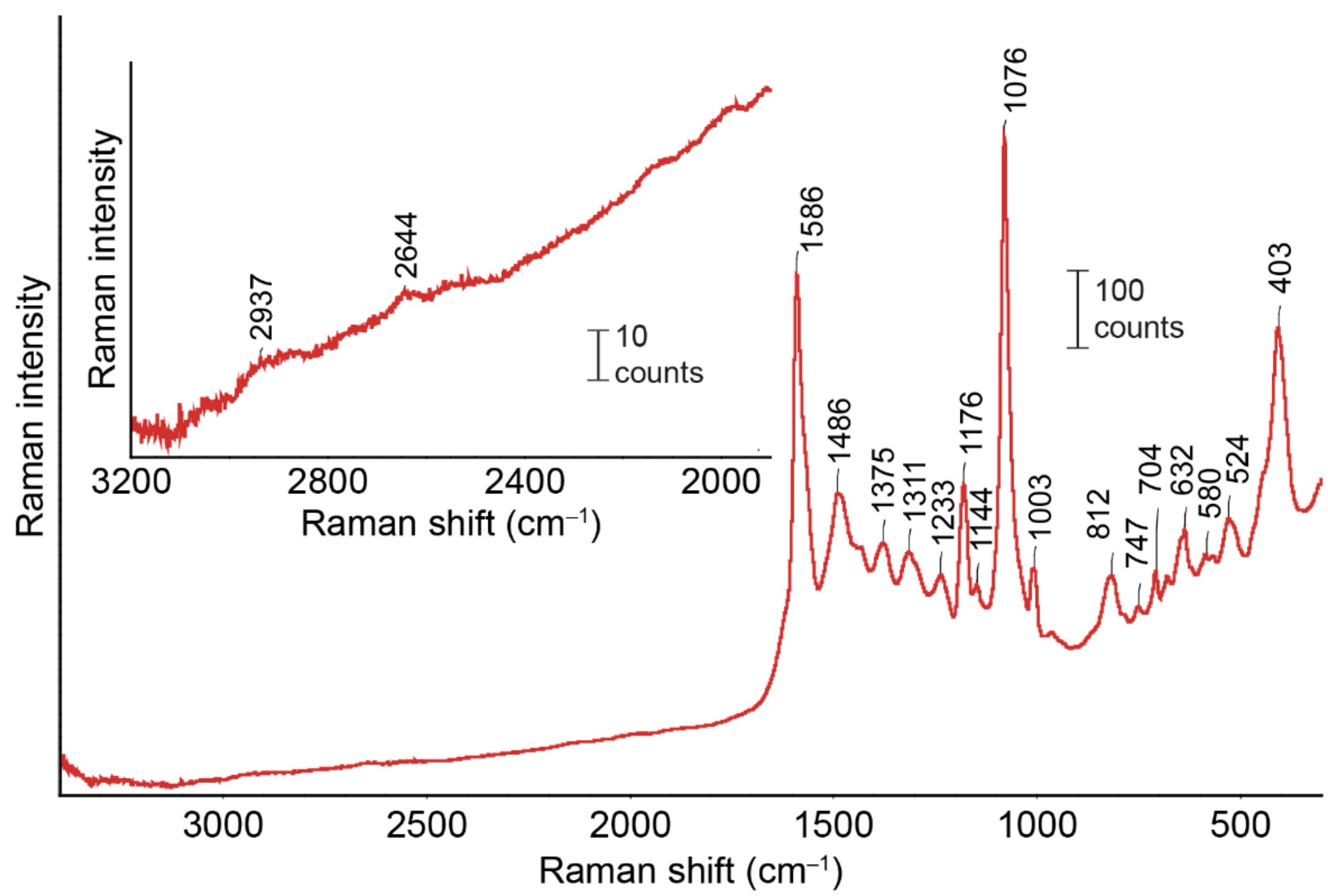

Figure S22 Au-SERS spectrum of 4-ABT/4,4′-DMAB measured with 780 nm excitation and obtained by averaging 10 individual spectra.

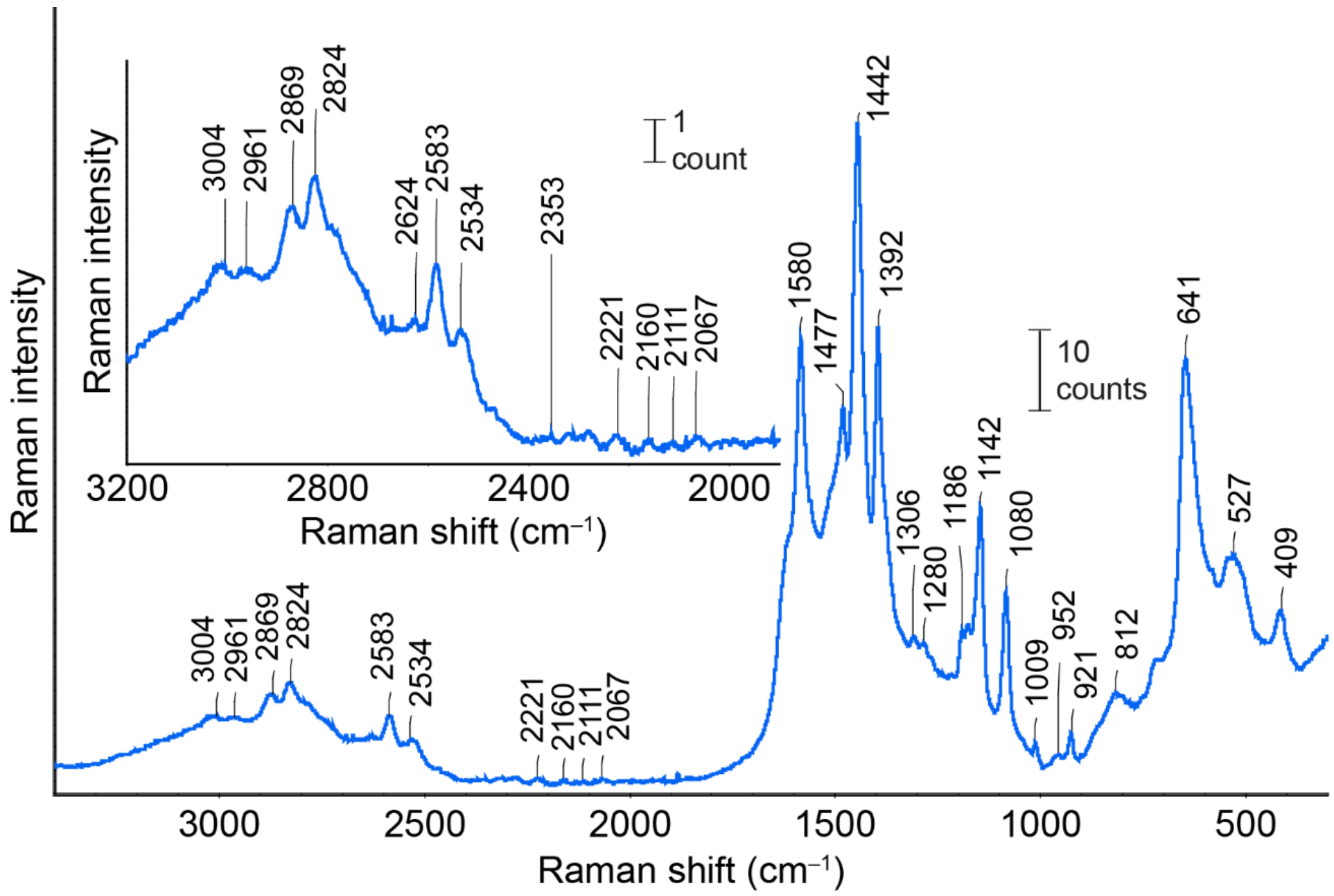

Figure S23 Cu-SERS spectrum of 4-ABT/4,4′-DMAB measured with 455 nm excitation and obtained by averaging 10 individual spectra.

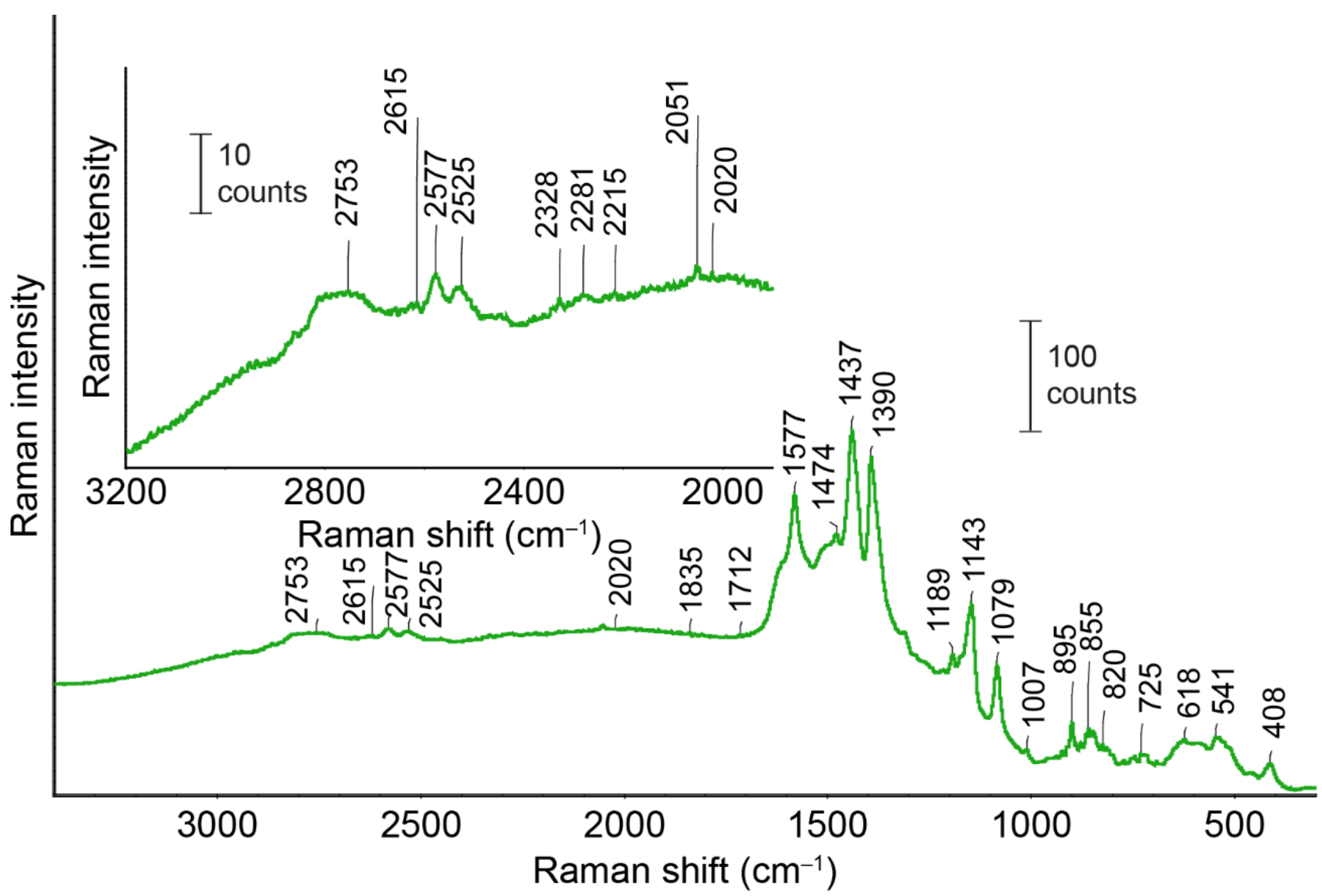

Figure S24 Cu-SERS spectrum of 4-ABT/4,4′-DMAB measured with 532 nm excitation and obtained by averaging 10 individual spectra.

Supplementary Note 1

A brief test using a different model probe, acetaminophen (AAP), was performed. Using the previously discussed silver large-scale enhancing substrate, we observed high-order transitions for excitation wavelengths of 532 and 633 nm (**Figures S25–S26**). Based on the interpretation presented above, this suggests that AAP is also capable of forming surface complexes with silver, likely involving the amine group, which has a known affinity for silver surfaces. This was supported by DFT simulations of absorption spectra for both native and silver-complexed AAP (**Figure S27**). While in the case of AAP some fundamental modes may also be observed in the 3200–2000 cm^{-1} region (namely those at 2925 and 2857 cm^{-1} in the 633 nm excited spectra, corresponding to C–H stretching vibrations), and are likely enhanced due to a favorable orientation of the aromatic ring parallel to the surface, which brings the methyl group closer to the metal surface, the presence of several additional bands attributable to high-order transitions is unambiguous.

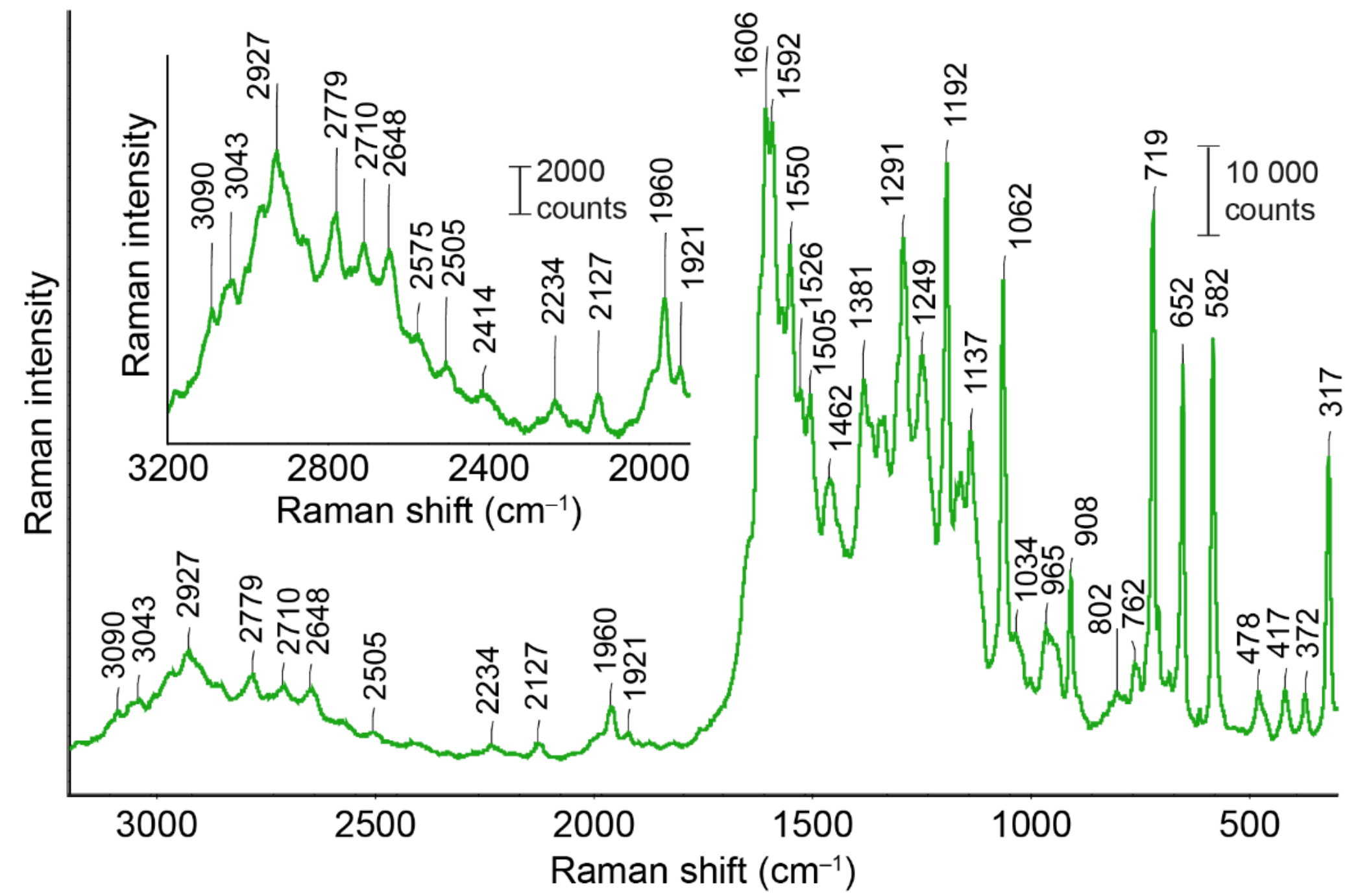

Figure S25 Ag-SERS spectrum of AAP and/or its products measured with 532 nm excitation.

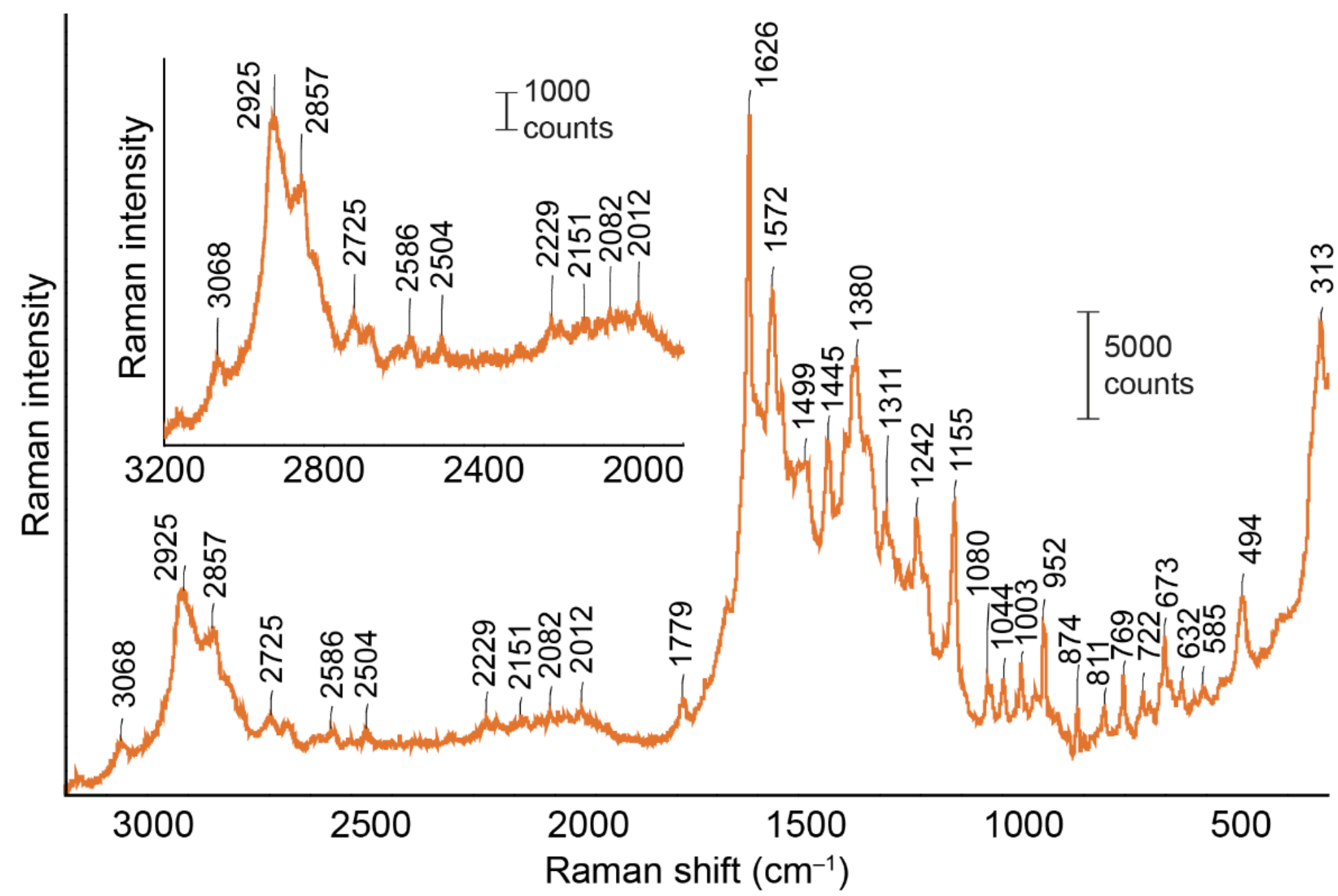

Figure S26 Ag-SERS spectrum of AAP and/or its products measured with 633 nm excitation.

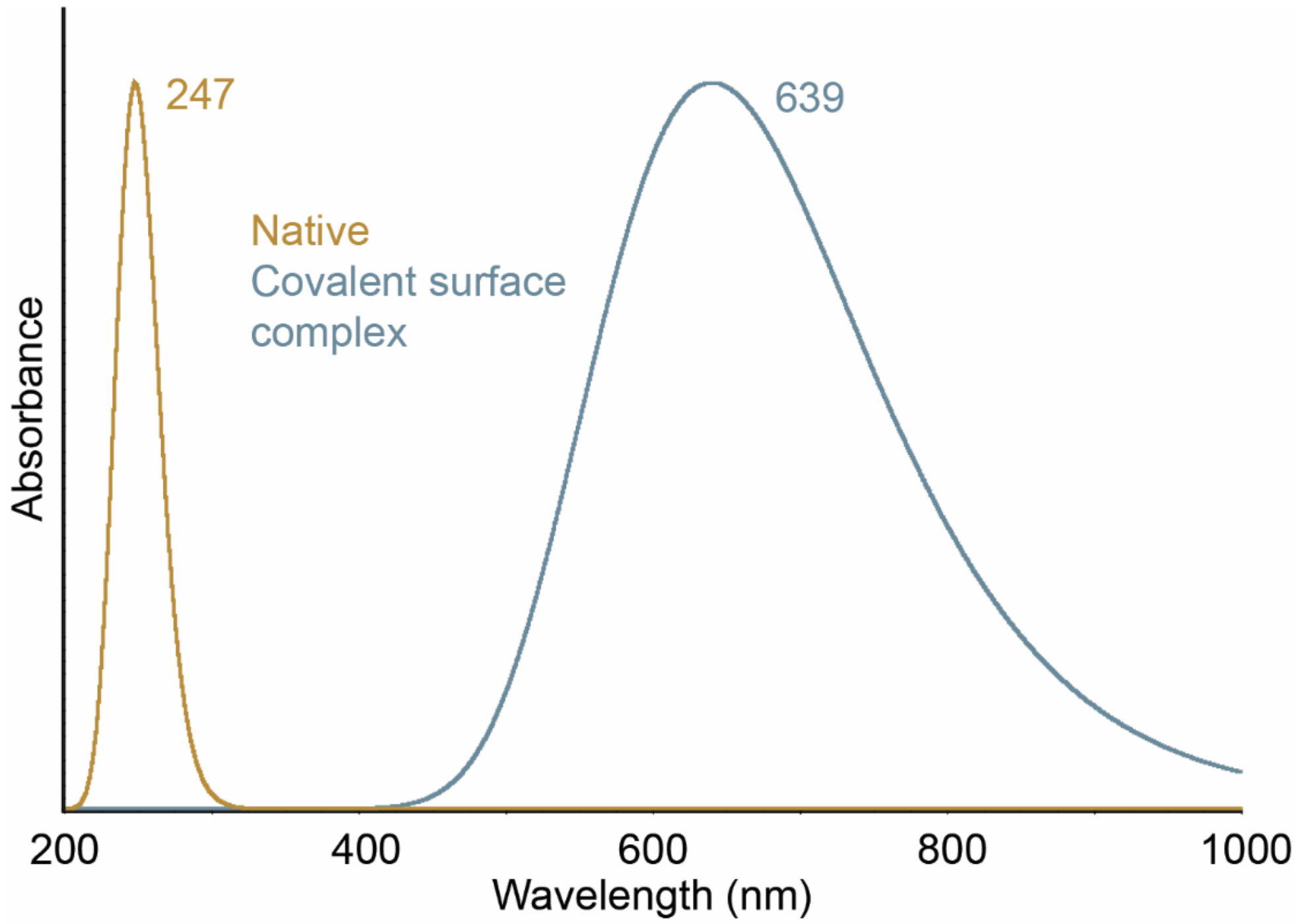

Figure S27 Calculated absorption spectra of AAP in its native state and covalently bound to a silver atom via the amine group.

Table S3 Experimental parameters used for Raman measurements of acetaminophen (AAP), benzenethiol (BT), and methylene blue (MB) for each excitation wavelength

Excitation wavelength (nm)	Mean resolution (cm^{-1})	Analyte	Laser power (μW)	Exposure number	Exposure time (s)
532	2	AAP	10.30	1	15
		BT	0.10	2	10
		MB	103.00	3	10
633	2	AAP	136.00	1	15
		BT	1.55	2	10
		MB	13.60	3	10
785	2	AAP	–	–	–
		BT	1.05	2	10
		MB	91.00	3	10
